# Investigating white matter alterations in Parkinson’s disease using multi-shell free-water DTI and NODDI: insights into neurodegeneration and levodopa effects

**DOI:** 10.3389/fneur.2025.1605753

**Published:** 2025-07-09

**Authors:** Maurizio Bergamino, Shuyi Zhu, Holly A. Shill, Ashley M. Stokes

**Affiliations:** ^1^Barrow Neuroimaging Innovation Center, Barrow Neurological Institute, Phoenix, AZ, United States; ^2^School of Life Sciences, Arizona State University, Tempe, AZ, United States; ^3^The Muhammad Ali Parkinson's Center, Barrow Neurological Institute, Phoenix, AZ, United States

**Keywords:** Parkinson’s disease, levodopa, diffusion MRI, white matter alterations, NODDI, free-water DTI

## Abstract

**Introduction:**

Parkinson’s disease (PD) is a progressive neurodegenerative disorder characterized by motor and non-motor symptoms. Levodopa remains the primary treatment, temporarily restoring dopamine levels and improving motor symptoms. Advanced diffusion MRI techniques, such as free-water corrected diffusion tensor imaging (fw-DTI) and neurite orientation dispersion and density imaging (NODDI), provide insights into PD-related microstructural changes beyond conventional DTI.

**Methods:**

This study investigates white matter alterations in PD using multi-shell fw-DTI and NODDI to compare voxel-wise differences between PD patients both OFF and ON levodopa, with comparison to healthy controls (HC). Effect sizes and receiver operating characteristic (ROC) analyses assessed the discriminative power of imaging metrics.

**Results:**

PD (OFF) exhibited increased free-water, reduced neurite density (NDI), and altered orientation dispersion (ODI) in key motor pathways in comparison to HC, while fw-FA offered robust group discrimination (AUC=0.956). Levodopa (ON state) increased NDI and NODDI-FWF, suggesting acute microstructural plasticity, though this finding contrasted with minimal fw-DTI FW changes. Additionally, voxel-based correlation analyses linked free-water and neurite integrity metrics with disease severity.

**Discussion:**

Our findings suggest that fw-DTI and NODDI provide complementary information on PD-related neurodegeneration and the transient effects of levodopa. These results underscore the potential of advanced diffusion MRI techniques as biomarkers for tracking PD progression and treatment response.

## 1 Introduction

Parkinson’s disease (PD) is the second most common neurodegenerative disorder after Alzheimer’s disease, affecting an estimated 6 million individuals worldwide in 2015, with projections suggesting this number may exceed 12 million by 2040 (1, 2). This progressive disease is primarily characterized by alpha-synuclein deposition in central and peripheral nervous system and degeneration of dopaminergic neurons in the substantia nigra pars compacta, leading to the hallmark motor manifestations of bradykinesia, resting tremor, rigidity, and postural instability (3–5). While the causes of PD remain unclear, the degeneration of dopaminergic neurons in the substantia nigra (SN) leads to decreased dopaminergic innervation in the striatum, which is a key factor in the pathophysiology of PD motor symptoms (4, 6).

Levodopa (L-DOPA) remains the most effective pharmacological treatment for PD, often providing marked improvement in motor function when patients are in the so-called therapeutically ON state (7). L-DOPA is able to cross the blood–brain barrier, where it is enzymatically converted into dopamine, temporarily restoring dopaminergic signaling, and improving motor symptoms (8). This mechanism directly targets the core pathophysiology of PD by replenishing dopamine levels in the striatum, a brain region essential for regulating motor function (9). The OFF and ON states experienced by patients reflect dynamic changes in dopaminergic signaling and can significantly impact a patient’s quality of life. Therefore, investigating the OFF and ON states is crucial for understanding disease progression, optimizing treatment regimens, and developing more targeted interventions.

In recent years, magnetic resonance imaging (MRI) has played an important role in studying the structural and functional changes associated with PD. Although conventional MRI sequences often fail to detect PD-specific alterations, especially in the early stages, advanced MRI techniques have shown promise in identifying microstructural and functional changes that correlate with disease severity and progression (10, 11). One advanced MRI method is diffusion MRI (dMRI), which is particularly sensitive to disruptions in the microstructural integrity of white matter by quantifying the diffusion of water molecules within brain tissue (12, 13).

The most commonly used dMRI technique is Diffusion Tensor Imaging (DTI), yielding metrics such as fractional anisotropy (FA) and mean diffusivity (MD). These biomarkers have been investigated in various regions affected by PD, including the substantia nigra, basal ganglia, and cortical areas (14, 15). Changes in these diffusion parameters have been connected to neurodegeneration and disease severity (16). While these biomarkers have proven effective in identifying chronic neurodegenerative changes, previous work has suggested a lack of dynamic differences between the OFF and ON states following L-DOPA administration (17).

Free-water DTI (fw-DTI) extends the standard DTI model to distinguish fw in extracellular spaces and water restricted within neural tissues. This differentiation is crucial for accurately assessing brain microstructure because the fw content in the brain can confound traditional DTI metrics (18). In the context of PD, fw-DTI has been used to identify microstructural white matter changes, with studies reporting elevated free-water levels in the posterior SN of PD patients that increase over time (14, 19). Furthermore, fw-DTI metrics have been shown to correlate with motor scores and cognition, as well as disease progression (20, 21).

Another advanced dMRI technique is Neurite Orientation Dispersion and Density Imaging (NODDI), which is designed to overcome some of the limitations of traditional DTI using multi-shell dMRI acquisitions to provide more specific metrics of microstructural complexity in the brain (22). NODDI involves a multi-compartment model that distinguishes intra-neurite, extra-neurite, and cerebrospinal fluid (CSF) contributions to the overall diffusion signal. Therefore, NODDI can estimate indices such as the Neurite Density Index (NDI) and Orientation Dispersion Index (ODI), which reflect the density and angular variability of neurites (axons and dendrites) within a brain voxel, respectively. Similar to FW-DTI, NODDI provides estimates of the free water fraction (FWF) inside the brain (22).

Previous studies have applied NODDI in PD to identify microstructural alterations in the substantia nigra and other nigrostriatal pathways—regions critically involved in PD pathology (23). For instance, reductions in the NDI may reflect decreased neurite density that is consistent with dopaminergic neuronal loss, while altered ODI might signify changes in neurite organization or orientation as the disease progresses (24, 25). Moreover, Wei et al. showed that NODDI metrics were significantly correlated with objective measures of gait in PD patients, particularly in the ON state (26).

In this study, multi-shell fw-DTI and NODDI were employed to investigate voxel-based microstructural differences in a cohort of 20 PD patients between their OFF and ON (levodopa) states. Additionally, voxel-wise differences were assessed between PD patients in the OFF state and a cohort of healthy controls (HC). For the PD group, voxel-based correlations were performed between clinical measures [MDS-Unified Parkinson’s Disease Rating Scale (27) and Hoehn and Yahr (H&Y) (28)] and fw-DTI/NODDI-derived metrics. This multimodal imaging approach may provide insights into how neurodegenerative processes and levodopa-induced changes influence brain microstructure, thereby enhancing the characterization of Parkinson’s disease pathology.

## 2 Methods

### 2.1 Participants

This study was performed in accordance with the local Institutional Review Board. Informed consent was obtained from all participants in this study, which was conducted in compliance with the Health Insurance Portability and Accountability Act (HIPAA). A total of 20 patients with non-demented, treated PD (25% female; mean age ± standard deviation (SD): 66.95 ± 8.86 years) and 16 healthy controls (HC) (43.75% female; mean age ± SD: 66.88 ± 7.44 years) were included in this study. All PD subjects were recruited by a movement disorders specialist, while HC were recruited through word-of-mouth referrals or our local volunteer database between September 2019 and November 2024.

Inclusion criteria required participants to be 40–90 years of age capable of providing informed consent, with PD patients maintaining stable antiparkinsonian medication and HCs being age-matched without neurological/psychiatric conditions. Exclusion criteria for both groups included MRI contraindications (bioimplants, aneurysm clips), severe claustrophobia/vertigo, marked tremor, or use of anticholinergic/dopamine-blocking drugs.

PD participants underwent motor assessment using the MDS-UPDRS-III, which evaluates motor symptoms such as rigidity, bradykinesia, tremor, gait, and postural stability through clinician-rated tasks. Each item is scored on a scale from 0 (normal) to 4 (severe impairment), with higher total scores indicating greater motor dysfunction (27). The H&Y scale was used to classify disease severity based on motor symptoms and functional impairment, ranging from mild unilateral involvement to severe disability requiring full-time assistance (28).

All study participants underwent MRI scanning, with HC subjects scanned once and PD subjects scanned twice, first in the OFF state (defined as >12 h since their last dose of levodopa) and subsequently in the ON state (practically defined as >1 h following levodopa administration). Following the OFF scans, PD patient received their typical morning dose of medication. Levodopa equivalent daily dose (LEDD) was calculated for all subjects^1^ (29).

### 2.2 MRI acquisition

Diffusion MRI data were acquired using a 3.0 T Philips Ingenia scanner. Imaging was performed using 2D Echo-Planar Imaging (EPI) with the following acquisition parameters: field of view (FOV) = 256 × 256 mm^2^, matrix size = 128 × 128, number of slices = 72, voxel size = 2.0 × 2.0 × 2.0 mm^3^, repetition time (TR) = 7,000 ms, echo time (TE) = 121 ms, and flip angle = 90°.

The multi-shell dMRI acquisition included one non-diffusion-weighted (*b* = 0 s/mm^2^, b0) image and four diffusion-weighted shells with b-values of 500, 1,000, 2,000, and 3,000 s/mm^2^, each acquired with 20 diffusion directions using phase-encoding along the antero-posterior (A/P) direction. Additionally, a *b* = 0 s/mm^2^ image with reversed phase-encoding polarity (posterior–anterior, P/A) was acquired to enable EPI distortion correction.

### 2.3 dMRI pre-processing

Diffusion MRI pre-processing was performed using MRtrix3 (version 3.0.4-145) (30), FSL (version 6.0.7.16) (31), and ANTs (version v2.4.4).^2^ The preprocessing pipeline included denoising using dwidenoise (MRtrix3) (32), EPI distortion correction, eddy current correction, and motion correction using TOPUP and eddy (FSL) (33). The eddy quality control tools were used to assess the quality of the DTI dataset (34). Slices with signal loss due to subject movement coinciding with diffusion encoding were identified and replaced using Gaussian process predictions (35). Subjects with more than 5% total outliers and/or an average absolute volume-to-volume head motion exceeding 3 mm were excluded.

Prior to MNI registration, the preprocessed dMRI native images in 2 mm were upsampled to 1.25 mm isotropic resolution using mrgrid (MRtrix3) (32) to reduce resolution mismatch and improve the accuracy of subsequent non-linear registration to the 1 mm MNI template using ANTs SyN. All brain extraction was performed on the upsampled b0 images using dwi2mask (MRtrix3) (36).

Subsequently, brain-extracted b0 images were non-linearly coregistered to the MNI standard space (1 × 1 × 1 mm) using the ANTs SyN registration algorithm (37). The resulting deformation fields were used to transform diffusion-derived maps into standard space for group-level analyses. Therefore, for each participant, fw-DTI and NODDI maps (see Free-Water Algorithm and NODDI sub-sections) were coregistered to MNI space by applying both the registration matrix and the warp field obtained from the b0 coregistration using ANTs. For the final voxel-based analysis, spatial smoothing with a 4 mm FWHM Gaussian kernel was applied to enhance signal-to-noise ratio and improve sensitivity to group differences, while maintaining a balance with spatial specificity relative to the native resolution.

### 2.4 Free water algorithm

The fw elimination model aims to mitigate the negative impact of the CSF partial volume effects on diffusion measurements (38). By distinguishing between freely moving water molecules and those that are hindered or restricted, this model provides a more accurate characterization of brain tissue microstructure. Multi-shell dMRI data were used for fw-DTI to overcome the known limitations of single-shell dMRI (39).

In this study, fw-corrected diffusion metrics, including fractional anisotropy (fw-FA) and the fw-index, were computed using the DIPY (version 1.10.0) (40) algorithm through the fwdti.FreeWaterTensorModel class and object and *fwdtimodel.fit* function. The multi-shell dMRI data were processed via an in-house Python (version 3.11.5) script.

### 2.5 Neurite orientation dispersion and density imaging (NODDI)

NODDI is an advanced dMRI model designed to provide a more detailed characterization of brain microstructure by distinguishing between different tissue compartments (22). Unlike conventional DTI, which assumes Gaussian diffusion, NODDI accounts for the complexity of neurite structures by modeling the diffusion signal as a combination of three compartments: the intra-neurite compartment, representing diffusion within axons and dendrites and characterized by restricted diffusion; the extra-neurite compartment, reflecting diffusion in the extracellular space where water diffusion is hindered by cellular structures; and the isotropic (fw) compartment, corresponding to unrestricted diffusion primarily associated with cerebrospinal fluid (CSF) and extracellular free water.

Using a multi-shell dMRI acquisition, NODDI estimates several key microstructural metrics including the neurite density index (NDI), which represents the volume fraction of the intra-neurite space and is an indicator of axonal and dendritic density; the orientation dispersion index (ODI), which quantifies the variability in neurite orientation and reflects the complexity of neural organization; and the isotropic volume fraction (FWF), which measures the fraction of freely diffusing water and is useful for detecting CSF contamination and neuroinflammation.

NODDI maps were generated using an in-house Python script implementing the Accelerated Microstructure Imaging via Convex Optimization (AMICO, version 2.0.3) framework (41), which enables efficient and robust estimation of NODDI metrics.^3^

### 2.6 Statistical analyses

The demographic and clinical characteristics, including age, sex, MDS-UPDRS-III, H&Y, motion, and outliers, are presented as means and standard deviations for each group.

Differences in age between groups were examined using a Student’s *t*-test, confirmed by the Shapiro–Wilk (SW) test for normality, while differences in sex were evaluated using the *χ*^2^ test.

Motor symptoms and disease severity for the PD group between OFF and ON conditions were analyzed using the paired *t*-test for the MDS-UPDRS-III test and the Wilcoxon Signed-Rank Test for the H&Y test (Table 1). Differences in tremor, bradykinesia, rigidity, and axial subscores between ON and OFF states were evaluated by the Wilcoxon Signed-Rank Test, with *p*-values reported as both uncorrected and corrected for multiple comparisons by Bonferroni’s method. Group differences in motion and outliers across groups or ‘OFF/ON’ status were analyzed using the Wilcoxon Rank-Sum Test, Wilcoxon Signed-Rank Test, and paired *t*-test. Complete information can be found in Table 1.

**Table 1:** Demographic, clinical, and motion characteristics of healthy controls (HC) and Parkinson’s disease (PD) patients in OFF and ON states.

	Control	ON	OFF
Group	HC	PD	PD
**Age**	66.88 (7.44)	66.95 (8.86)	–
Shapiro Wilk test	*W* = 0.96; *p* = 0.6889	*W* = 0.92; *p* = 0.103	–
*t-test*	*t* = −0.028; *p* = 0.978		–
**# (%F)**	16 (43.75%)	20 (25.00%)	–
*χ* ^2^	*χ*^2^ = 38.45; *p* < 0.001*		–
**MDS-UPDRS-III (total)**	–	26.55 (11.87)	14.30 (6.94)
Shapiro Wilk test	–	*W* = 0.95; *p* = 0.327	*W* = 0.93; *p* = 0.142
*Paired t-test*	–	*t* = 6.717; *p* < 0.001*	
**Hoehn and Yahr Stage**	–	1.70 (0.73)	1.65 (0.59)
Shapiro Wilk test	–	*W* = 0.84; *p* = 0.004*	*W* = 0.74; *p* < 0.001*
*Wilcoxon Signed-Rank Test*	–	*V* = 4.0; *p* = 0.773	

Motion during dMRI acquisitions			
Group	HC	PD	PD
**ABS Motion (mm)**	0.62 (0.28)	0.52 (0.20)	0.59 (0.22)
Shapiro Wilk test	*W* = 0.81; *p* = 0.004*	*W* = 0.96; *p* = 0.484	*W* = 0.95; *p* = 0.359
*Wilcoxon Rank-Sum Test - HC vs.*		PD_on_: *W* = 152; *p* = 0.811	PD_off_: *W* = 187; *p* = 0.399
*paired t-test (PD_off_ vs. PD_on_)*		*t* = −1.566; *p* = 0.134	
**REL Motion (mm)**	0.28 (0.08)	0.28 (0.11)	0.29 (0.14)
Shapiro Wilk test	*W* = 0.86; *p* = 0.018*	*W* = 0.92; *p* = 0.090	*W* = 0.86; *p* = 0.007*
*Wilcoxon Rank-Sum Test - HC vs.*		PD_on_: *W* = 170; *p* = 0.762	PD_off_: *W* = 158; *p* = 0.962
*Wilcoxon Signed-Rank Test (PD_off_ vs. PD_on_)*		*V* = 97; *p* = 0.784	
**Outlier (%)**	0.06 (0.25)	0.08 (0.27)	0.06 (0.28)
Shapiro Wilk test	*W* = 0.27; *p* < 0.001*	*W* = 0.236; *p* < 0.001*	*W* = 0.24; *p* < 0.001*
*Wilcoxon Rank-Sum Test - HC vs.*	*W* = 154; *p* = 0.715	PD_on_: *W* = 161.5; *p* = 0.936	PD_off_: *W* = 154; *p* = 0.715
*Wilcoxon Signed-Rank Test (PD_off_ vs. PD_on_)*		*V* = 2.0; *p* = 1.000	

MDS-UPDRS-III (sections)	ON	OFF	Wilcoxon Signed–Rank test
Axial	0.62 (0.80)	0.35 (0.61)	*V* = 313.5; *p* < 0.001; p_corr_ < 0.001
Bradykinesia	1.08 (0.94)	0.61 (0.78)	*V* = 7,921; *p* < 0.001; p_corr_ < 0.001
Rigidity	0.84 (0.83)	0.42 (0.64)	*V* = 991.5; *p* < 0.001; p_corr_ < 0.001
Tremor	0.53 (0.75)	0.26 (0.56)	*V* = 1,400; *p* < 0.001; p_corr_ < 0.001

Results of statistical analyses, including Shapiro–Wilk tests for normality, *t*-tests, and Wilcoxon rank-sum and signed-rank tests, are presented for each variable. Data are shown as mean (standard deviation, SD) or percentages, with significant findings indicated (*p < 0.05). Motor symptom evaluation of Parkinson’s disease patients in OFF and ON states based on MDS-UPDRS-III sections. Mean values (standard deviations, SD) for each motor symptom are presented alongside results of the Wilcoxon signed-rank test for paired comparisons. Significant differences between OFF and ON states are indicated by the Wilcoxon Signed-Rank Test (p_corr_: p corrected for multiple comparisons by Bonferroni’s).

HC, healthy control; PD, Parkinson’s disease; F, females, *χ*^2^, chi-squared test; ABS Motion, absolute motion; REL motion, relative motion.

Voxel-based analyses, including between-group comparisons [HC *vs.* PD (OFF)], ‘OFF/ON’ status assessments, and correlations with cognitive test scores, were restricted to voxels within the combined masks derived from the ICBM-DTI-81 white matter labels atlas and the JHU white matter tractography atlas (42, 43). Statistically significant clusters were identified and annotated based on these atlases.

To evaluate voxel-based differences between HC and PD (OFF) across all diffusion-related metrics, a Student’s *t*-test was performed using a linear model. Age, sex, and average absolute motion were included as covariates to control for potential confounding factors. For within-subject comparisons between PD (OFF) and PD (ON), a paired Student’s *t*-test was applied, with average absolute motion as covariate. Additionally, for the PD group (both OFF and ON conditions), voxel-based correlations between diffusion metrics and cognitive scores were computed using a linear model, adjusting for average absolute motion. All analyses were conducted using an in-house Python script.

For voxel-based statistical analyses, the Threshold-Free Cluster Enhancement (TFCE) method was employed to enhance cluster detection while avoiding arbitrary thresholding and addressing multiple comparisons (44). Additionally, a Family-Wise Error (FWE) correction at the 0.05 level was applied using the Benjamini-Hochberg procedure (45) (FDR < 0.05). Effect sizes were computed for all analyses using Hedges’ *g* (46), where |*g*| ≥ 1.03 and |*g*| ≥ 0.61 indicated a large effect size for differences between HC and PD (OFF) and differences between PD (OFF) and PD (ON), respectively (both at *α* = 0.05, power = 0.85). For correlations with motor symptoms and disease severity, Spearman’s correlation coefficient (*ρ*) (47) was used, along with the corresponding *t* value.

To assess the discriminative power of NODDI and fw-DTI metrics, we computed the area under the receiver operating characteristic curve (AUC) for each metric. The AUC distributions were estimated using a nonparametric bootstrap resampling approach with 10,000 iterations. This method provides robust estimates of AUC variability by resampling the data with replacement, generating empirical distributions that account for the uncertainty in AUC estimation.

For each metric, we computed the mean AUC along with 95% confidence intervals (CIs) derived from the bootstrap distribution. The confidence intervals were estimated using the bias-corrected and accelerated (BCa) bootstrap method to account for potential skewness in the AUC distribution.

Additionally, to statistically compare the performance of different metrics, pairwise differences in AUC values were computed using the bootstrap method. The differences in AUCs were calculated for all metric pairs, and corresponding 95% confidence intervals were derived. A statistically significant difference was inferred when the confidence interval did not include zero, indicating a meaningful distinction in classification performance between the metrics.

All AUC related analyses were performed using R [version 4.4.2 (2024-10-31)] and RStudio (version 2024.09.0) by using the pROC and boot packages.

## 3 Results

For the PD cohort, 19 participants received levodopa, with 7 of them also taking amantadine, 3 taking rasagiline, and 2 taking ropinirole. One participant was just on a combination of amantadine and rasagiline. The mean time between the last medication dose and the OFF scan was 12.8 ± 0.1 h, while the mean time between drug administration and the ON scan was 68.9 ± 6.0 min. The mean disease duration was 5.5 ± 2.9 years. Demographic and clinical characteristics of the recruited individuals are summarized in Table 1, while disease duration and medications are summarized for PD participants in Table 2.

**Table 2:** Medication specifics and disease duration of the PD cohort.

Disease duration	# subjects	Mean LEDD	Number meds (avg)
<5 yrs	7	188.1	1.7
5–10 yrs	12	239.9	2.2
>10 yrs	1	154.0	3.0

LEDD, Levodopa Equivalent Daily Dose; meds, medications.

No significant difference in age was found between HC and PD (*t* = −0.028, *p* = 0.978). However, a significant difference was observed between the two groups for sex with males more common in the PD group (*χ*^2^ = 38.45, *p* < 0.001).

For within-subject comparisons between PD (OFF) and PD (ON), a significant difference was found for MDS-UPDRS-III (*t* = 6.717, *p* < 0.001), while no significant difference was observed for the H&Y score (*V* = 4.0, *p* = 0.773).

No significant differences were detected in the dMRI-derived motion parameters, including absolute motion, relative motion, or total outliers, across groups or between the OFF and ON stages. In all final analyses, no subjects were excluded due to motion or outlier effects. All statistical results for cognitive scores, motion, and outliers are summarized in Table 1.

Table 1 shows tremor, bradykinesia, rigidity, and axial subscores for PD patients in ON and OFF states based on MDS-UPDRS-III sub-sections. Significant statistical differences between the OFF and ON stages were found for all subscores (*p* < 0.001).

### 3.1 fw-DTI results: HC vs. PD (OFF)

**Figure 1:**
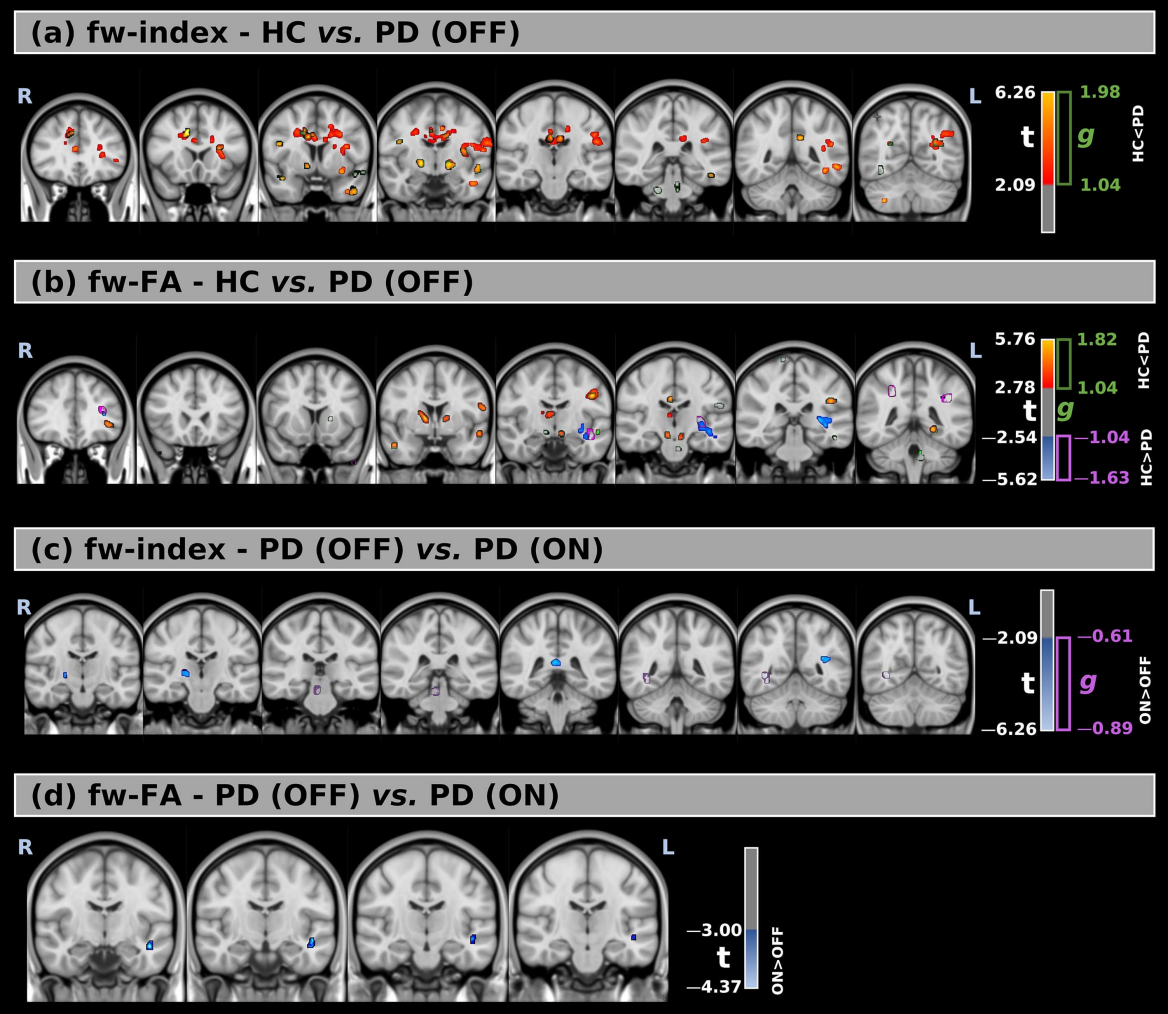
**(a,b)** Voxel-wise comparison of free-water index (fw-index) and free-water–corrected fractional anisotropy (fw-FA) between healthy controls (HC) and Parkinson’s Disease (PD) patients in the OFF medication state. **(c,d)** Comparison of fw-index and fw-FA between PD patients in the OFF and ON states. Statistical maps are overlaid on MNI-space anatomical images. Significant group differences (FWE-corrected *p* < 0.05) are displayed using red-to-yellow color scales for increased values in PD (*t* > 0) and blue-to-light blue for increased values in HC or PD ON (*t* < 0). Clusters with large effect sizes are outlined in green (g positive) and purple (g negative).

The voxel-wise analysis identified statistical differences between HC and PD (OFF) in fw-index and fw-FA metrics (Figures 1a,b, Table 3(a), and Supplementary Figure 1).

**Table 3:** (a) Results of the voxel-based analysis for fw-DTI between HC and PD (OFF). The analysis includes fw-FA and the fw-index. Results are reported as statistical values (*t*-values) and effect sizes. The percentage (%) represents the volume of the cluster within the corresponding white matter location based on the atlas. (b) Results of the voxel-based analysis for fw-DTI between PD (OFF) and PD (ON). The analysis includes fw-FA and the fw-index. Results are reported as statistical values (*t*-values) and effect sizes. The percentage (%) represents the volume of the cluster within the corresponding white matter location based on the atlas.

(a)								
fw-FA	HC<PD (OFF)				HC>PD (OFF)			
	*t*-tests		Effect-size		Two-sample *t*-tests		Effect-size	
	vol (%)	*t*	vol (%)	*g*	vol (%)	*t*	vol (%)	*g*
JHU Atlas								
Anterior Thalamic Radiation L	0.75	3.570	1.33	1.136	0.40	−4.057	0.41	−1.236
Anterior Thalamic Radiation R	2.37	3.455	1.85	1.190	–	–	–	–
Cortical spinal tract L	–	–	0.32	1.077	–	–	–	–
Cortical spinal tract R	–	–	0.27	1.102	–	–	–	–
Cingulum Hippo L	1.15	4.156	1.54	1.167	–	–	–	–
Forceps Major	–	–	0.32	1.144	–	–	0.27	−1.143
Inferior fronto-occipital fasc L	–	–	0.24	1.111	4.37	−3.252	1.32	−1.183
Inferior Longitudinal fasc L	–	–	–	–	2.40	−3.130	0.70	−1.137
Inferior Longitudinal fasc R	0.32	4.051	0.57	1.186	–	–	–	–
Superior Longitudinal fasc L	1.91	3.697	0.97	1.156	0.33	−3.396	0.38	−1.149
Uncinate fasc L	–	–	0.32	1.123	0.80	−3.570	0.50	−1.246
Sup Longitudinal fasc temporal L	3.28	3.742	1.61	1.138	0.60	−3.301	–	–
ICBM81 Atlas								
Middle cerebellar peduncle	–	–	0.23	1.133	–	–	–	–
Body of corpus callosum	0.36	3.959	0.53	1.177	–	–	–	–
Corticospinal tract L	–	–	2.63	1.075	–	–	–	–
Inferior cerebellar peduncle L	–	–	3.82	1.233	–	–	–	–
Cerebral peduncle R	3.34	3.680	6.28	1.180	–	–	–	–
Cerebral peduncle L	4.61	3.756	7.07	1.167	–	–	–	–
Anterior limb of internal capsule R	9.08	3.532	5.13	1.204	–	–	–	–
Anterior limb of internal capsule L	2.62	3.400	4.24	1.121	–	–	–	–
Posterior limb of internal capsule R	2.53	3.178	0.37	1.044	–	–	–	–
Retrolenticular part of internal capsule L	–	–	–	–	22.56	−3.345	4.82	−1.174
Sagittal stratum L	–	–	–	–	11.61	−2.869	–	–
External capsule L	–	–	–	–	2.29	−2.824	–	–
Fornix (cres)/Stria terminalis L	–	–	–	–	0.53	−2.725	–	–

fw-index	HC<PD (OFF)			
	Two–sample *t*-tests		Effect-size	
	vol (%)	*t*	vol (%)	*g*
JHU Atlas				
Anterior Thalamic Radiation L	1.16	2.774	0.52	1.126
Anterior Thalamic Radiation R	0.53	4.102	0.56	1.309
Cingulum cingulate gyrus L	1.22	3.304	1.03	1.118
Cingulum cingulate gyrus R	17.53	2.920	4.65	1.128
Cingulum Hippo R	0.28	3.981	0.25	1.258
Forceps Major	0.38	3.123	0.48	1.093
Forceps Minor	1.75	3.215	0.68	1.303
Inferior fronto-occipital fasc L	1.92	2.744	0.26	1.069
Inferior Longitudinal fasc L	2.79	3.128	1.04	1.086
Inferior Longitudinal fasc R	–	–	0.85	1.085
Superior Longitudinal fasc L	6.41	2.779	0.50	1.098
Superior Longitudinal fasc R	0.55	3.496	0.55	1.185
Uncinate fasc L	1.73	2.776	–	–
Uncinate fasc R	0.30	3.753	0.33	1.100
Sup Longitudinal fasc temporal L	6.30	2.888	0.53	1.097
ICBM81 Atlas				
Middle cerebellar peduncle	0.22	4.039	0.81	1.128
Pontine crossing tract (a part of MCP)	–	–	2.73	1.071
Genu of corpus callosum	0.44	2.252	–	–
Body of corpus callosum	22.84	2.651	4.46	1.143
Splenium of corpus callosum	1.23	2.758	0.53	1.079
Medial lemniscus L	–	–	11.59	1.089
Cerebral peduncle L	0.70	3.526	0.44	1.134
Anterior limb of internal capsule L	0.89	2.433	–	–
Posterior limb of internal capsule R	1.57	3.720	1.23	1.125
Posterior limb of internal capsule L	1.63	3.608	0.91	1.119
Anterior corona radiata R	2.32	2.677	–	–
Anterior corona radiata L	7.44	2.678	0.85	1.088
Superior corona radiata R	11.89	2.555	0.64	1.134
Superior corona radiata L	9.20	2.404	–	–
Posterior corona radiata R	0.32	3.118	–	–
Posterior corona radiata L	3.07	2.900	0.94	1.055
Posterior thalamic radiation R	0.30	3.180	–	–
Posterior thalamic radiation L	2.56	2.665	0.28	1.057
External capsule L	2.95	2.951	–	–
Cingulum (cingulate gyrus) R	14.77	2.829	3.76	1.089
Cingulum (cingulate gyrus) L	0.47	3.362	–	–
Superior longitudinal fasciculus R	0.30	3.056	–	–
Superior longitudinal fasciculus L	5.98	2.840	0.89	1.129
Superior fronto-occipital fasciculus L	1.58	2.345	–	–

(b)				
fw-FA	PD (OFF) < PD (ON)			
	*t*-tests		Effect-size	
JHU Atlas	vol (%)	*t*	vol (%)	*g*
Inferior Longitudinal fasc L	0.41	−3.475	–	–

fw-index	PD (OFF) < PD (ON)			
	*t*-tests		Effect-size	
	vol (%)	*t*	vol (%)	*g*
JHU Atlas				
Forceps Major	0.22	−3.893	–	–
Sup Longitudinal fasc temporal L	0.21	−3.726	–	–
ICBM81 Atlas				
Splenium of corpus callosum	0.57	−3.904	–	–
Superior cerebellar peduncle R	–	–	9.88	−0.693
Posterior limb of internal capsule R	0.53	−3.383	–	–
Posterior thalamic radiation R	–	–	2.84	−0.686
Sagittal stratum R	–	–	2.69	−0.675
Superior longitudinal fasciculus L	0.35	−3.750	–	–

*t*, *t*-value; *g*, Hedges’ *g* (effect-size); vol, volume.

The fw-index was significantly higher in PD (OFF) than in HC, with statistical differences across large portions of the white matter. Additionally, fw-FA was also elevated in PD (OFF) in several white matter regions, including the right and left anterior thalamic radiation (*t* = 3.57, *g* = 1.14; *t* = 3.46, *g* = 1.19, respectively), left superior longitudinal fasciculus (*t* = 3.74, *g* = 1.14), right and left cerebral peduncle (*t* = 3.68, *g* = 1.18; *t* = 3.76, *g* = 1.17, respectively), and the right and left anterior limb of the internal capsule (*t* = 3.53, *g* = 1.20; *t* = 3.40, *g* = 1.12, respectively).

Conversely, fw-FA was significantly lower in PD (OFF) than HC in a large cluster within the left retrolenticular part of the internal capsule (*t* = −3.35, *g* = 1.17). The differences between the two groups were also supported by effect size (with large effect-size values) for both metrics.

### 3.2 fw-DTI results: PD (OFF) vs. PD (ON)

Figures 1c,d, Table 3 (b), and Supplementary Figure 1 present the voxel-based statistical differences between PD (OFF) and PD (ON). We observed higher values for both fw-related metrics in the ON state compared to the OFF state. However, these differences were limited to small clusters (cluster volume less than 1% of the corresponding white matter area). Additionally, these findings were not supported by the effect size analysis.

### 3.3 NODDI results: HC vs. PD (OFF)

**Figure 2:**
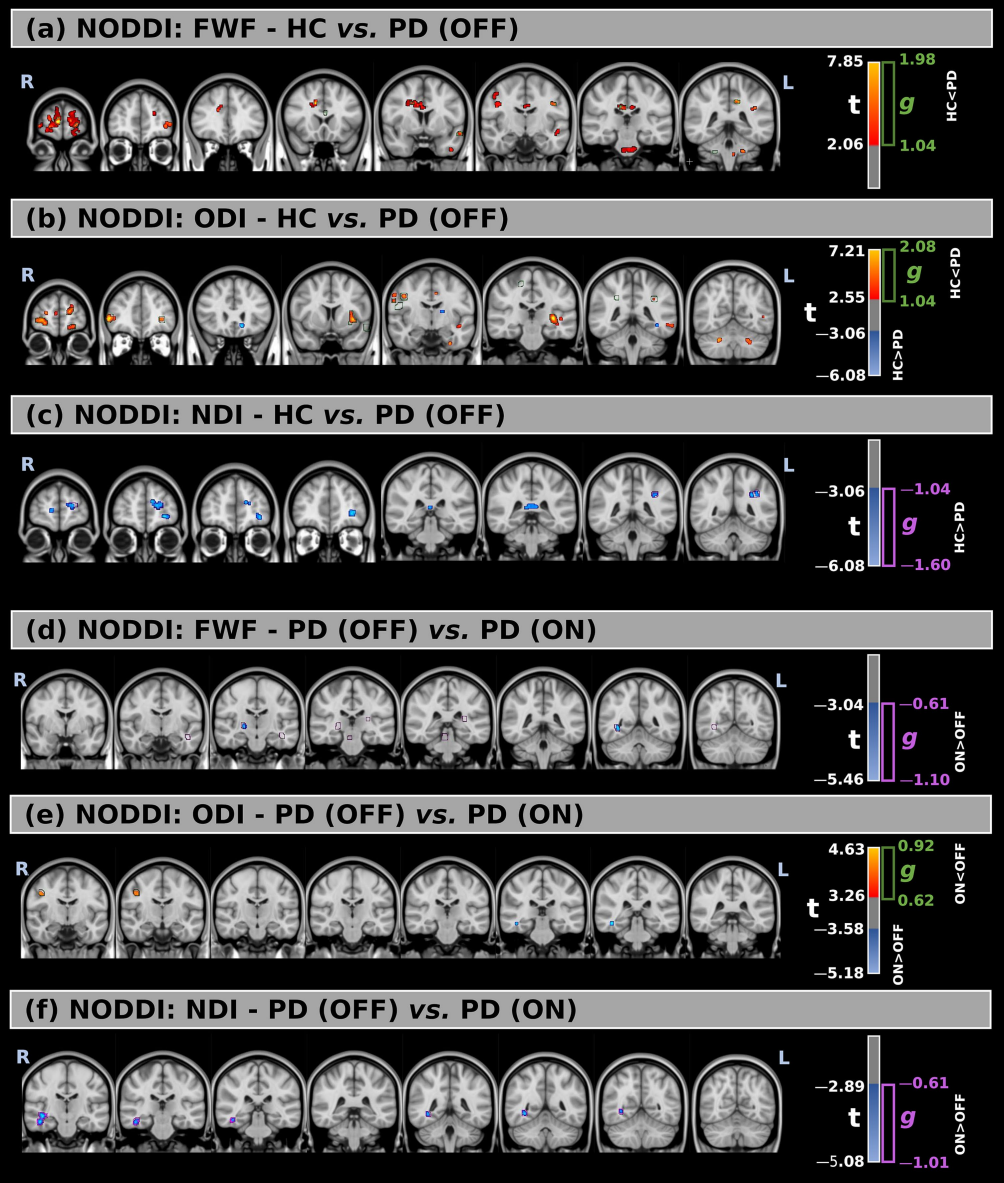
**(a–c)** Voxel-wise comparison of NODDI-derived metrics between healthy controls (HC) and Parkinson’s Disease (PD) patients in the OFF medication state, including free-water fraction (FWF), orientation dispersion index (ODI), and neurite density index (NDI), respectively. **(d–f)** Comparison of the same NODDI metrics between PD patients in the OFF and ON states. Statistical group differences are overlaid on MNI-space anatomical images. Significant regions (*p* < 0.05, FWE-corrected) are shown using red-to-yellow color scales for positive *t*-values and blue-to-light blue for negative *t*-values. Green and purple contours indicate clusters with large effect sizes.

This study analyzed three NODDI-derived metrics: FWF, ODI, and NDI. Figures 2a–c, Table 4, and Supplementary Figure 2 present the voxel-based differences between HC and PD (OFF) patients for these metrics.

**Table 4:** Results of the voxel-based analysis for NODDI metrics between HC and PD (OFF).

Atlas	NODDI - FWF				NODDI - ODI						NODDI - NDI			
	HC<PD (OFF)				HC<PD (OFF)				HC>PD (OFF)		HC>PD (OFF)			
	*t*-tests		Effect-size		*t*-tests		Effect-size		*t*-tests		*t*-tests		Effect-size	
	vol (%)	*t*	vol (%)	*g*	vol (%)	*t*	vol (%)	*g*	vol (%)	*t*	vol (%)	*t*	vol (%)	*g*
JHU Atlas														
Anterior Thalamic Radiation L	1.42	2.682	–	–	0.79	3.251	0.64	1.196	0.34	−3.409	0.55	−3.101	0.09	−1.156
Anterior Thalamic Radiation R	0.61	2.452	–	–	0.77	3.345	0.27	1.097	–	–	–	–	–	–
Cortical spinal tract L	2.25	2.646	–	–	0.22	3.412	–	–	–	–	–	–	–	–
Cortical spinal tract R	0.56	2.479	–	–	0.27	3.754	0.65	1.188	–	–	–	–	–	–
Cingulum cingulate gyrus R	4.31	2.857	–	–	–	–	–	–	–	–	–	–	–	–
Forceps Major	–	–	–	–	–	–	–	–	–	–	0.60	−3.202	–	–
Forceps Minor	4.53	2.826	0.45	1.462	3.01	3.430	1.01	1.222	0.25	−3.368	0.73	−3.150	0.13	−1.168
Inferior fronto-occipital fasc L	–	–	–	–	3.64	3.321	1.83	1.188	0.23	−3.462	0.45	−3.150	–	–
Inferior fronto-occipital fasc R	–	–	–	–	0.36	3.282	–	–	–	–	–	–	–	–
Inferior Longitudinal fasc L	0.37	2.961	–	–	1.31	3.171	0.43	1.172	0.18	−3.493	–	–	–	–
Inferior Longitudinal fasc R	–	–	0.16	1.094	–	–	–	–	0.21	−4.365	–	–	–	–
Superior Longitudinal fasc L	0.77	3.076	0.16	1.136	0.39	3.442	0.34	1.145	–	–	–	–	0.15	−1.164
Superior Longitudinal fasc R	0.68	2.728	–	–	0.39	3.887	0.80	1.175	–	–	–	–	–	–
Uncinate fasc L	–	–	–	–	1.72	3.572	1.43	1.172	0.22	−3.443	0.51	−3.222	–	–
Sup Longitudinal fasc temporal L	0.59	3.331	0.18	1.134	0.52	3.314	–	–	–	–	–	–	–	–
Sup Longitudinal fasc temporal R	0.61	2.937	–	–	0.38	3.690	0.92	1.211	–	–	–	–	–	–
ICBM81 Atlas														
Middle cerebellar peduncle	1.66	2.685	0.25	1.201	1.69	3.508	–	–	–	–	–	–	–	–
Pontine crossing tract (a part of MCP)	2.47	2.683	–	–	–	–	–	–	–	–	–	–	–	–
Genu of corpus callosum	–	–	–	–	–	–	–	–	1.45	−3.493	–	–	–	–
Body of corpus callosum	4.78	2.678	0.68	1.127	–	–	–	–	**–**	**–**	–	–	–	–
Splenium of corpus callosum	–	–	–	–	–	–	–	–	**–**	**–**	1.48	−3.172	–	–
Corticospinal tract R	6.02	2.455	–	–	–	–	–	–	**–**	**–**	–	–	–	–
Corticospinal tract L	12.04	2.700	–	–	–	–	–	–	**–**	**–**	–	–	–	–
Medial lemniscus L	3.43	3.128	–	–	–	–	–	–	**–**	**–**	–	–	–	–
Inferior cerebellar peduncle L	0.72	3.126	–	–	–	–	–	–	**–**	**–**	–	–	–	–
Anterior limb of internal capsule L	–	–	–	–	–	–	–	–	0.86	−3.470	–	–	–	–
Posterior limb of internal capsule L	–	–	–	–	–	–	–	–	1.12	−3.346	–	–	–	–
Retrolenticular part of internal capsule L	–	–	–	–	14.74	3.569	7.05	1.224	**–**	**–**	–	–	–	–
Anterior corona radiata R	0.79	2.516	–	–	–	–	–	–	**–**	**–**	–	–	–	–
Anterior corona radiata L	–	–	–	–	–	–	–	–	0.54	−3.479	–	–	–	–
Superior corona radiata R	4.47	2.496	0.44	1.166	–	–	–	–	**–**	**–**	–	–	–	–
Superior corona radiata L	0.59	2.905	–	–	–	–	–	–	**–**	**–**	–	–	–	–
Posterior thalamic radiation R	–	–	–	–	–	–	–	–	1.36	−4.341	–	–	–	–
Sagittal stratum L	–	–	–	–	8.56	2.913	0.63	1.039	2.33	−3.493	–	–	–	–
External capsule L	–	–	–	–	3.17	3.267	1.75	1.130	**–**	**–**	–	–	–	–
Cingulum (cingulate gyrus) R	1.67	2.623	–	–	–	–	–	–	**–**	**–**	–	–	–	–

Results are reported as statistical values (*t*-values) and effect sizes. The percentage (%) represents the volume of the cluster within the corresponding white matter location based on the atlas.

*t*, *t*-value; *g*, Hedges’ *g* (effect-size); vol, volume; FWF, Free Water Fraction; ODI, Orientation Dispersion Index; NDI, Neurite Density Index; HC, healthy control; PD, Parkinson’s disease.

Comparisons between the two groups revealed significant differences across all metrics. FWF values were higher in PD (OFF) compared to HC in multiple white matter regions, with large effect sizes observed mainly in the forceps minor (*t* = 2.83, *g* = 1.46), left superior longitudinal fasciculus (*t* = 3.08, *g* = 1.14), middle cerebellar peduncle (*t* = 2.69, *g* = 1.20), the body of the corpus callosum (*t* = 2.68, *g* = 1.13), and right superior corona radiata (*t* = 2.50, *g* = 1.17).

For ODI, significant increases in PD (OFF) were detected in multiple white matter regions, with the largest significant clusters located in the left retrolenticular part of the internal capsule (*t* = 3.57, *g* = 1.22) and the left sagittal stratum (*t* = 2.91, *g* = 1.04). Additionally, several smaller clusters showed reduced ODI values in PD (OFF) compared to HC in different white matter locations. However, they were not confirmed by the effect size at a large effect level.

Lastly, NDI was significantly lower in PD (OFF) compared to HC, with large effect sizes confirming this difference mainly inside the left anterior thalamic radiation (*t* = −3.10, *g* = −1.16) and forceps minor (*t* = −3.15, *g* = −1.17).

### 3.4 NODDI: PD (OFF) vs. PD (ON)

Figures 2d–f, Table 5, and Supplementary Figure 2 show the voxel-based statistical differences in NODDI-related metrics between PD patients in the OFF and ON medication states.

**Table 5:** Results of the voxel-based analysis for NODDI metrics between PD (OFF) and PD (ON).

Atlas	NODDI - FWF				NODDI - ODI						NODDI - NDI			
	PD (ON) > PD (OFF)				PD (ON) < PD (OFF)				PD (ON) > PD (OFF)		PD (ON) > PD (OFF)			
	*t*-tests		Effect-size		*t*-tests		Effect-size		*t*-tests		*t*-tests		Effect-size	
	vol (%)	*t*	vol (%)	*g*	vol (%)	*t*	vol (%)	*g*	vol (%)	*t*	vol (%)	*t*	vol (%)	*g*
JHU Atlas														
Anterior Thalamic Radiation R	–	–	0.62	−0.668	–	–	–	–	–	–	–	–	–	–
Cortical spinal tract R	0.41	−3.634	0.60	−0.661	–	–	–	–	–	–	–	–	–	–
Forceps Minor	–	–	0.61	−0.726	–	–	–	–	–	–	–	–	–	–
Inferior fronto-occipital fasc L	0.40	−3.445	0.72	−0.732	–	–	–	–	–	–	–	–	–	–
Inferior fronto-occipital fasc R	–	–	0.56	−0.707	–	–	–	–	0.11	−4.220	1.00	−3.561	0.92	−0.812
Inferior Longitudinal fasc L	–	–	0.26	−0.678	–	–	–	–	–	–	–	–	–	–
Inferior Longitudinal fasc R	0.19	−4.146	0.44	−0.691	–	–	–	–	0.25	−4.169	2.36	−3.608	1.89	−0.724
Superior Longitudinal fasc R	–	–	–	–	0.25	3.798	0.18	0.712	–	–	–	–	–	–
Uncinate fasc L	0.86	−3.432	1.21	−0.740	–	–	–	–	–	–	–	–	–	–
Uncinate fasc R	–	–	0.22	−0.715	–	–	–	–	–	–	–	–	–	–
Sup Longitudinal fasc temporal R	–	–	–	–	0.61	3.881	0.46	0.724	–	–	–	–	–	–
ICBM81 Atlas														
Genu of corpus callosum	–	–	0.69	−0.755	–	–	–	–	–	–	–	–	**–**	**–**
Superior cerebellar peduncle R	–	–	9.68	−0.661	–	–	–	–	–	–	–	–	**–**	**–**
Posterior limb of internal capsule R	1.76	−3.652	1.47	−0.658	–	–	–	–	–	–	–	–	**–**	**–**
Retrolenticular part of internal capsule R	–	–	0.60	−0.660	–	–	–	–	–	–	–	–	**–**	**–**
Retrolenticular part of internal capsule L	–	–	2.27	−0.690	–	–	–	–	–	–	–	–	**–**	**–**
Anterior corona radiata R	–	–	0.82	−0.724	–	–	–	–	–	–	–	–	**–**	**–**
Posterior thalamic radiation R	0.55	−4.252	1.59	−0.683	–	–	–	–	–	–	0.93	−3.340	0.52	−0.814
Sagittal stratum R	0.76	−4.085	1.48	−0.709	–	–	–	–	2.02	−4.198	8.93	−3.601	5.33	−0.631
Sagittal stratum L	–	–	0.63	−0.658	–	–	–	–	–	–	–	–	**–**	**–**
External capsule R	–	–	–	–	–	–	–	–	–	–	0.46	−3.273	0.73	−0.727

Results are reported as statistical values (*t*-values) and effect sizes. The percentage (%) represents the volume of the cluster within the corresponding white matter location based on the atlas.

*t*, *t*-value; *g*, Hedges’ *g* (effect-size); vol, volume; FWF, Free Water Fraction; ODI, Orientation Dispersion Index; NDI, Neurite Density Index; HC, healthy control; PD, Parkinson’s disease.

FWF values were higher in the ON state compared to the OFF state across several white matter regions, with large effect sizes observed mainly in the right corticospinal tract (*t* = −3.63, *g* = −0.66), left inferior fronto-occipital fasciculus (*t* = −3.45, *g* = −0.73), left uncinate fasciculus (*t* = −3.43, *g* = −0.74), right posterior limb of the internal capsule (*t* = −3.65, *g* = −0.66), right posterior thalamic radiation (*t* = −4.25, *g* = −0.68), and right sagittal stratum (*t* = −4.09, *g* = −0.71).

Only minimal differences were detected between the OFF and ON states in both statistical directions for ODI. However, these findings were not supported by large effect sizes.

Finally, compared to the OFF state, higher NDI values were observed in the ON state within the right inferior fronto-occipital fasciculus (*t* = −3.56, *g* = −0.81), right inferior longitudinal fasciculus (*t* = −3.61, *g* = −0.72), and right sagittal stratum (*t* = −3.60, *g* = −0.63).

### 3.5 fw-DTI metrics and correlations with MDS-UPDRS III/H&Y scores

**Figure 3:**
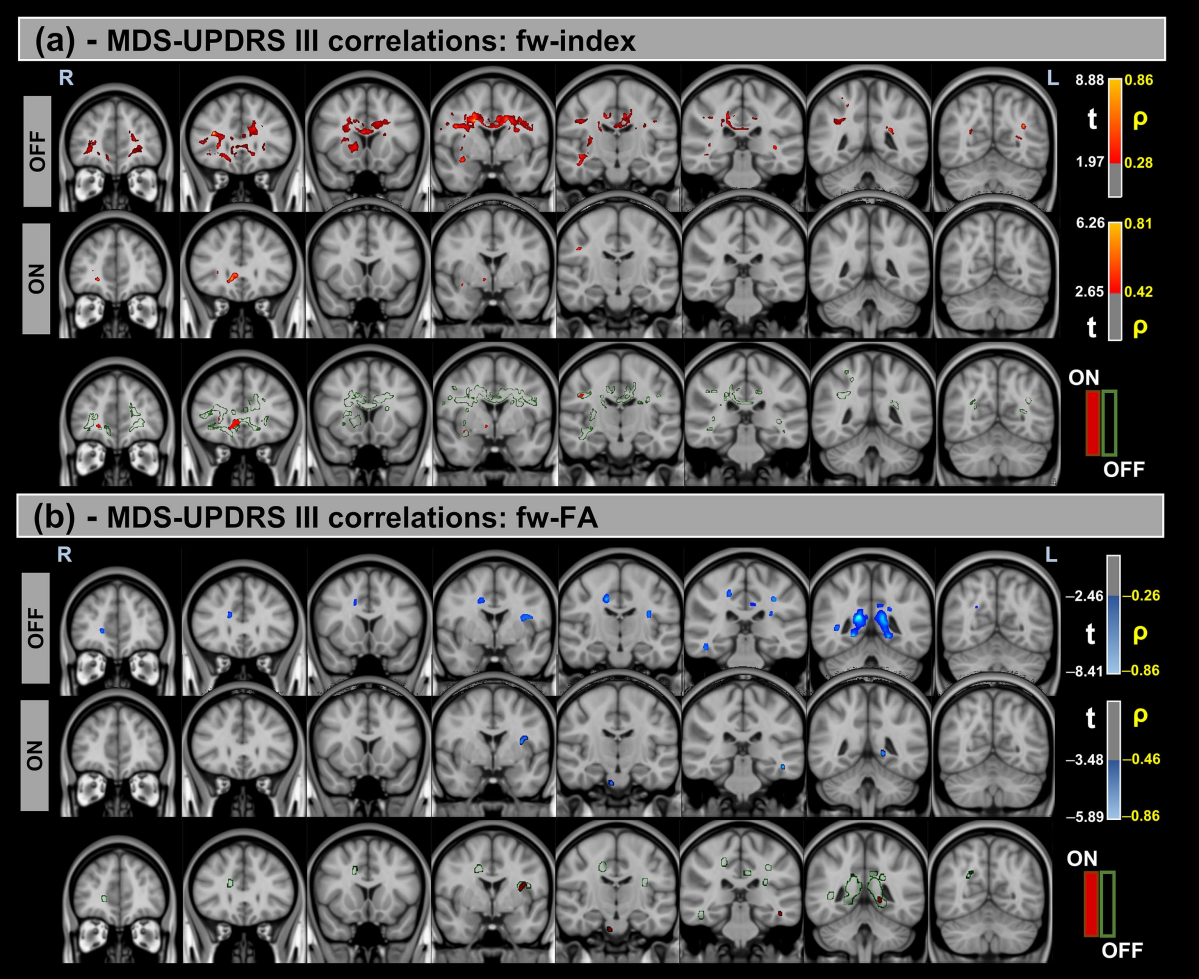
Voxel-based correlations between fw-DTI metrics and MDS-UPDRS-III scores in the Parkinson’s Disease (PD) group. **(a)** Positive correlations with the fw-index are shown warm colors (red-yellow). **(b)** Negative correlations with the fw-FA index are shown cool colors (blue-light blue). The color bars represent *t*-values and corresponding correlation coefficients (*ρ*). Differences between OFF and ON states (green for OFF and red for ON) indicate a reduction in correlation strength during the ON state.

**Figure 4:**
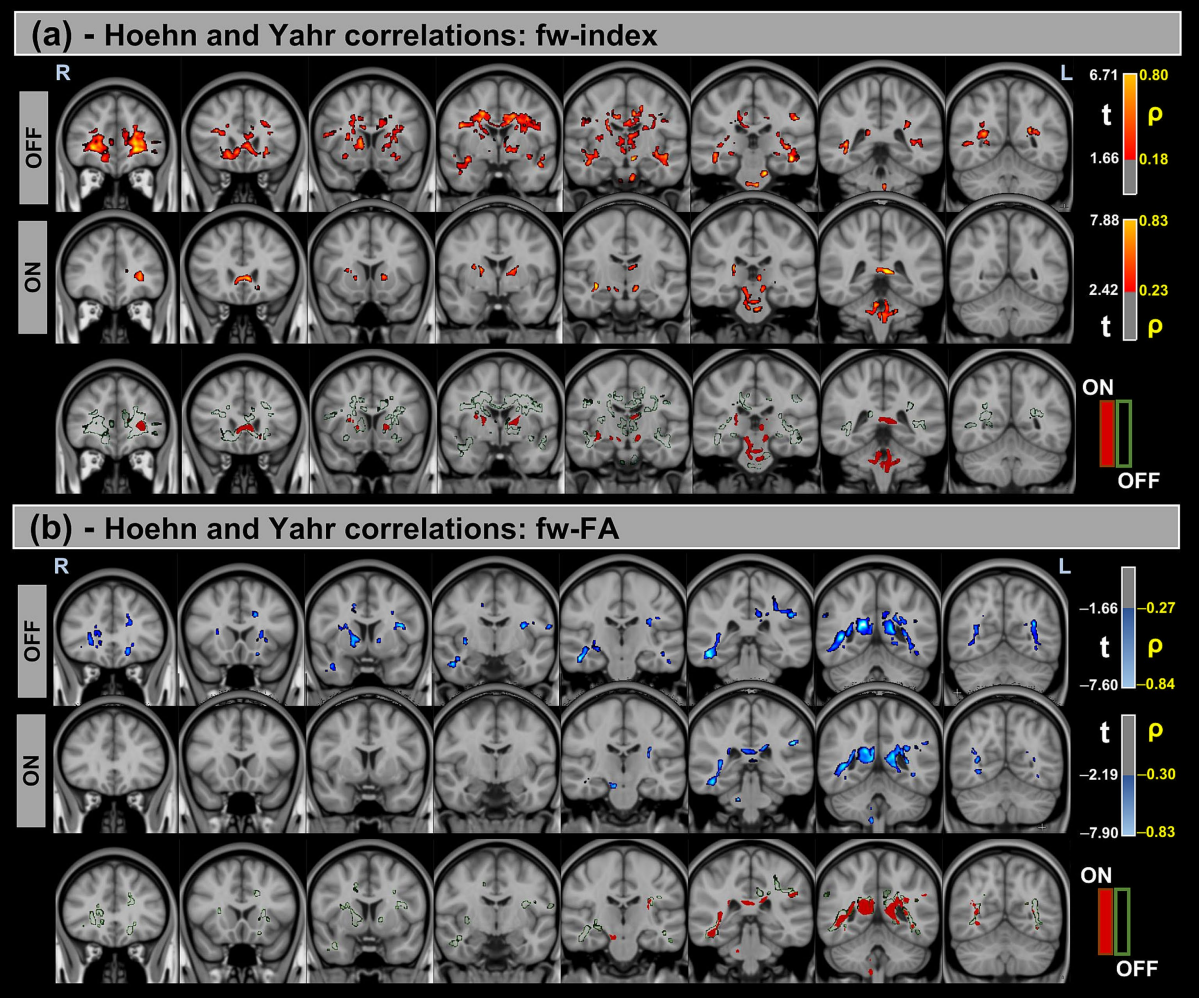
Voxel-based correlations between fw-DTI metrics and H&Y scores in the Parkinson’s Disease (PD) group. **(a)** Positive correlations with the fw-index are shown warm colors (red-yellow). **(b)** Negative correlations with the fw-FA index are shown cool colors (blue-light blue). The color bars represent *t*-values and corresponding correlation coefficients (*ρ*). Differences between OFF and ON states (green for OFF and red for ON) indicate the variation in correlation strength during the ON state.

Figures 3, 4 show voxel-based correlations between fw-DTI metrics and MDS-UPDRS-III/H&Y in the PD group OFF and ON states.

Significant positive correlations were observed between the fw-index and MDS-UPDRS-III in both OFF and ON states, though these correlations were reduced during the ON state. Conversely, significant negative correlations were found between the fw-FA index and MDS-UPDRS-III, with a similar reduction in correlation strength observed in the ON state compared to OFF. For both metrics, a prominent statistical cluster was identified within the splenium of the corpus callosum.

Similarly, significant positive correlations were detected between the fw-index and H&Y scores in both OFF and ON states. In contrast, significant negative correlations were found between fw-FA and H&Y across both conditions. Differences in correlation strength were also observed between the OFF and ON states.

Table 6 provides a summary of these findings, including cluster volumes, *t*-values, *ρ*-values, and the percentage change in volume between the OFF and ON states.

**Table 6:** Voxel-based correlations between fw-DTI metrics and clinical scores (MDS-UPDRS-III and Hoehn and Yahr) in PD groups during the OFF and ON stages.

Atlas	MDS-UPDRS III							Hoehn and Yahr						
	fw-FA - PD (OFF)			fw-FA - PD (ON)			Δ vol% PD (ON) – PD (OFF)	fw-FA - PD (OFF)			fw-FA - PD (ON)			Δ vol% PD (ON) – PD (OFF)
	vol (%)	*t*	*ρ*	vol (%)	*t*	*ρ*	Δvol (%)	vol (%)	*t*	*ρ*	vol (%)	*t*	*ρ*	Δvol (%)
JHU Atlas														
Anterior Thalamic Radiation L	–	–	–	–	–	–	–	4.58	−2.472	−0.466	0.15	−2.831	−0.481	−4.43
Anterior Thalamic Radiation R	0.36	−3.321	−0.568	–	–	–	−0.36	6.12	−2.484	−0.471	0.41	−3.607	−0.579	−5.72
Cortical spinal tract L	0.47	−3.777	−0.495	–	–	–	−0.47	0.57	−2.416	−0.461	0.93	−3.781	−0.534	0.36
Cortical spinal tract R	0.41	−3.169	−0.633	0.05	−3.862	−0.657	−0.37	–	–	–	2.26	−3.359	−0.508	2.26
Cingulum cingulate gyrus L	2.90	−2.884	−0.566	–	–	–	−2.90	11.16	−2.399	−0.459	0.04	−3.032	−0.589	−11.13
Cingulum cingulate gyrus R	1.55	−2.909	−0.356	–	–	–	−1.55	0.53	−1.997	−0.429	0.57	−2.580	−0.495	0.05
Cingulum Hippo L	8.94	−3.268	−0.488	0.71	−3.760	−0.598	−8.23	3.94	−2.329	−0.484	4.41	−2.970	−0.504	0.47
Cingulum Hippo R	8.94	−3.186	−0.425	–	–	–	−8.94	2.62	−2.735	−0.532	1.83	−2.812	−0.566	−0.79
Forceps Major	7.64	−3.837	−0.567	0.43	−4.262	−0.763	−7.22	14.67	−2.556	−0.499	18.40	−3.525	−0.543	3.73
Forceps Minor	0.96	−3.382	−0.549	–	–	–	−0.96	6.07	−2.388	−0.490	–	–	–	−6.07
Inferior fronto-occipital fasc L	–	–	–	0.22	−3.877	−0.745	0.22	11.17	−2.346	−0.482	2.03	−3.058	−0.542	−9.14
Inferior fronto-occipital fasc R	0.79	−3.592	−0.621	0.14	−4.536	−0.769	−0.65	16.76	−2.322	−0.472	8.09	−2.931	−0.507	−8.68
Inferior Longitudinal fasc L	–	–	–	0.28	−3.726	−0.731	0.28	8.96	−2.240	−0.467	2.60	−3.026	−0.534	−6.36
Inferior Longitudinal fasc R	1.06	−3.673	−0.614	0.07	−4.364	−0.745	−0.99	20.60	−2.521	−0.498	10.08	−2.931	−0.528	−10.52
Superior Longitudinal fasc L	1.18	−3.745	−0.505	0.06	−3.725	−0.708	−1.12	5.79	−2.440	−0.472	2.45	−3.146	−0.509	−3.33
Superior Longitudinal fasc R	0.49	−3.873	−0.627	–	–	–	−0.49	4.21	−2.637	−0.507	1.90	−2.848	−0.487	−2.31
Uncinate fasc L	–	–	–	–	–	–	–	9.60	−2.505	−0.507	–	–	–	−9.60
Uncinate fasc R	–	–	–	–	–	–	–	7.03	−2.176	−0.452	–	–	–	−7.03
Sup Longitudinal fasc temporal L	0.64	−3.518	−0.558	0.05	−3.891	−0.723	−0.59	4.92	−2.403	−0.474	2.42	−3.352	−0.504	−2.50
Sup Longitudinal fasc temporal R	1.52	−3.909	−0.632	–	–	–	−1.52	11.48	−2.690	−0.514	5.89	−2.851	−0.482	−5.58
ICBM81 Atlas														
Middle cerebellar peduncle	–	–	–	0.42	−3.912	−0.669	0.42	–	–	–	2.29	−3.932	−0.631	2.29
Genu of corpus callosum	0.32	−3.420	−0.554	–	–	–	−0.32	5.51	−2.062	−0.455	–	–	–	−5.51
Body of corpus callosum	1.95	−3.277	−0.523	–	–	–	−1.95	1.15	−2.411	−0.496	0.24	−2.517	−0.488	−0.91
Splenium of corpus callosum	24.03	−3.686	−0.567	–	–	–	−24.03	34.17	−2.673	−0.510	43.38	−3.465	−0.538	9.22
Corticospinal tract R	–	–	–	–	–	–	–	–	–	–	0.44	−2.762	−0.500	0.44
Medial lemniscus R	–	–	–	–	–	–	–	–	–	–	10.14	−3.815	−0.599	10.14
Medial lemniscus L	–	–	–	–	–	–	–	–	–	–	0.29	−3.389	−0.620	0.29
Inferior cerebellar peduncle R	–	–	–	–	–	–	–	4.65	−3.136	−0.541	17.87	−3.781	−0.675	13.22
Inferior cerebellar peduncle L	–	–	–	–	–	–	–	–	–	–	6.51	−3.824	−0.680	6.51
Cerebral peduncle R	–	–	–	–	–	–	–	–	–	–	11.55	−3.206	−0.460	11.55
Cerebral peduncle L	1.80	−4.059	−0.584	–	–	–	−1.8	–	–	–	–	–	–	–
Anterior limb of internal capsule R	–	–	–	–	–	–	–	23.93	−2.701	−0.478	–	–	–	−23.93
Anterior limb of internal capsule L	–	–	–	–	–	–	–	10.34	−2.580	−0.393	–	–	–	−10.34
Posterior limb of internal capsule R	–	–	–	–	–	–	–	1.65	−2.069	−0.391	–	–	–	−1.65
Posterior limb of internal capsule L	0.24	−3.461	−0.443	–	–	–	−0.24	0.21	−2.074	−0.253	0.64	−3.519	−0.474	0.43
Retrolenticular part of internal capsule R	–	–	–	–	–	–	–	18.37	−2.293	−0.452	19.17	−2.950	−0.440	0.80
Retrolenticular part of internal capsule L	–	–	–	–	–	–	–	0.85	−2.884	−0.505	1.22	−3.699	−0.476	0.36
Anterior corona radiata R	5.37	−3.368	−0.547	–	–	–	−5.37	26.28	−2.263	−0.470	–	–	–	−26.28
Anterior corona radiata L	–	–	–	–	–	–	–	17.28	−2.544	−0.509	–	–	–	−17.28
Superior corona radiata R	4.21	−3.252	−0.549	–	–	–	−4.21	1.67	−2.446	−0.487	–	–	–	−1.67
Superior corona radiata L	1.04	−3.477	−0.440	–	–	–	−1.04	1.66	−2.314	−0.466	0.44	−3.497	−0.512	−1.23
Posterior corona radiata R	0.72	−3.168	−0.643	–	–	–	−0.72	1.98	−2.445	−0.519	2.44	−2.522	−0.458	0.46
Posterior corona radiata L	1.97	−3.242	−0.461	–	–	–	−1.97	9.88	−2.347	−0.473	5.41	−3.392	−0.514	−4.47
Posterior thalamic radiation R	1.61	−3.827	−0.666	–	–	–	−1.61	32.23	−2.272	−0.476	23.79	−2.800	−0.534	−8.43
Posterior thalamic radiation L	–	–	–	–	–	–	–	24.38	−2.164	−0.452	8.12	−2.923	−0.528	−16.26
Sagittal stratum R	3.50	−3.472	−0.618	–	–	–	−3.5	57.00	−2.548	−0.512	29.67	−2.822	−0.537	−27.33
Sagittal stratum L	–	–	–	–	–	–	–	18.20	−2.622	−0.531	6.59	−3.163	−0.555	−11.61
External capsule R	–	–	–	–	–	–	–	13.30	−2.287	−0.463	–	–	–	−13.30
External capsule L	1.24	−3.580	−0.465	–	–	–	−1.24	2.63	−2.423	−0.471	0.16	−3.394	−0.420	−2.47
Cingulum (cingulate gyrus) R	3.20	−3.014	−0.551	–	–	–	−3.2	5.29	−2.088	−0.469	5.55	−3.246	−0.601	0.26
Cingulum (cingulate gyrus) L	6.43	−2.964	−0.589	–	–	–	−6.43	6.80	−2.026	−0.417	0.91	−2.666	−0.499	−5.89
Cingulum (hippocampus) R	4.21	−2.839	−0.420	–	–	–	−4.21	0.89	−1.803	−0.410	3.56	−3.142	−0.618	2.67
Cingulum (hippocampus) L	2.60	−2.841	−0.372	–	–	–	−2.6	0.43	−1.913	−0.355	1.21	−2.575	−0.489	0.78
Fornix (cres)/Stria terminalis R	0.71	−3.283	−0.452	–	–	–	−0.71	13.70	−1.996	−0.403	–	–	–	−13.70
Superior longitudinal fasciculus R	1.15	−3.861	−0.615	–	–	–	−1.15	4.25	−2.798	−0.518	1.86	−2.802	−0.454	−2.39
Superior longitudinal fasciculus L	1.53	−3.606	−0.542	–	–	–	−1.53	5.81	−2.196	−0.449	2.33	−3.248	−0.517	−3.48
Uncinate fasciculus R	–	–	–	–	–	–	–	2.37	−1.896	−0.396	–	–	–	−2.37
Tapetum R	–	–	–	–	–	–	–	50.34	−2.431	−0.487	53.86	−3.102	−0.544	3.52
Tapetum L	–	–	–	–	–	–	–	39.67	−2.469	−0.516	49.00	−3.321	−0.597	9.33

Atlas	MDS-UPDRS III							Hoehn and Yahr						
	fw-index - PD (off)			fw-index - PD (on)			Δ vol% PD (on) - PD (off)	fw-index - PD (off)			fw-index - PD (on)			Δ vol% PD (on) - PD (off)
	vol (%)	*t*	*ρ*	vol (%)	*t*	*ρ*	Δvol (%)	vol (%)	*t*	*ρ*	vol (%)	*t*	*ρ*	Δvol (%)
JHU Atlas														
Anterior Thalamic Radiation L	8.20	3.010	0.516	–	–	–	−8.20	20.67	2.374	0.469	11.90	3.059	0.539	−8.77
Anterior Thalamic Radiation R	11.77	2.651	0.501	2.75	3.636	0.674	−9.02	18.01	2.255	0.457	5.66	3.135	0.586	−12.34
Cortical spinal tract L	0.38	2.190	0.262	–	–	–	−0.38	3.41	2.702	0.509	5.72	3.275	0.616	2.32
Cortical spinal tract R	1.53	2.313	0.372	0.17	3.447	0.663	−1.35	2.57	2.252	0.424	6.68	3.059	0.601	4.11
Cingulum cingulate gyrus L	25.77	2.675	0.462	–	–	–	−25.77	17.29	2.228	0.450	0.74	2.796	0.343	−16.55
Cingulum cingulate gyrus R	7.37	2.565	0.544	–	–	–	−7.37	7.57	2.139	0.449	–	–	–	−7.57
Cingulum Hippo R	0.00	0.000	0.000	–	–	–	–	2.50	1.951	0.443	–	–	–	−2.50
Forceps Major	2.79	3.541	0.582	–	–	–	−2.79	10.58	2.377	0.450	1.57	4.486	0.650	−9.01
Forceps Minor	14.66	2.615	0.521	1.75	3.271	0.602	−12.91	18.16	2.283	0.456	2.78	3.157	0.557	−15.38
Inferior fronto-occipital fasc L	8.15	3.171	0.555	0.43	4.100	0.671	−7.72	20.72	2.451	0.482	1.82	2.925	0.413	−18.89
Inferior fronto-occipital fasc R	16.30	2.794	0.509	2.80	3.455	0.668	−13.50	22.98	2.271	0.450	0.72	3.649	0.608	−22.27
Inferior Longitudinal fasc L	2.14	3.398	0.575	0.49	4.091	0.663	−1.65	10.01	2.289	0.469	–	–	–	−10.01
Inferior Longitudinal fasc R	7.13	3.221	0.560	0.04	3.963	0.736	−7.09	16.34	2.275	0.455	–	–	–	−16.34
Superior Longitudinal fasc L	3.15	2.812	0.479	0.02	4.738	0.642	−3.13	6.74	2.248	0.454	–	–	–	−6.74
Superior Longitudinal fasc R	10.80	2.660	0.472	0.18	3.796	0.583	−10.62	6.20	2.180	0.445	0.36	3.211	0.378	−5.84
Uncinate fasc L	13.34	3.159	0.556	0.00	0.000	0.000	−13.34	23.59	2.521	0.486	4.32	2.930	0.407	−19.27
Uncinate fasc R	20.88	2.736	0.522	5.80	3.503	0.666	−15.08	36.10	2.321	0.468	0.16	3.560	0.547	−35.94
Sup Longitudinal fasc temporal L	3.86	2.810	0.469	–	–	–	−3.86	7.88	2.180	0.445	–	–	–	−7.88
Sup Longitudinal fasc temporal R	12.04	2.711	0.460	0.03	3.539	0.513	−12.01	10.11	2.060	0.428	0.28	3.185	0.355	−9.83
ICBM81 Atlas														
Middle cerebellar peduncle	–	–	–	–	–	–	–	1.53	2.754	0.542	2.29	2.913	0.483	0.76
Pontine crossing tract (a part of MCP)	–	–	–	–	–	–	–	0.73	2.520	0.487	54.40	3.269	0.628	53.67
Genu of corpus callosum	30.09	2.590	0.540	5.06	3.194	0.569	−25.03	23.82	2.177	0.458	10.90	3.167	0.576	−12.91
Body of corpus callosum	49.81	2.849	0.539	–	–	–	−49.81	31.54	2.166	0.446	–	–	–	−31.54
Splenium of corpus callosum	7.85	2.633	0.517	–	–	–	−7.85	8.04	2.302	0.459	3.86	4.459	0.649	−4.18
Corticospinal tract R	–	–	–	–	–	–	–	8.30	2.727	0.479	4.70	2.806	0.568	−3.60
Corticospinal tract L	–	–	–	–	–	–	–	16.86	2.908	0.540	3.36	3.168	0.638	−13.50
Medial lemniscus R	–	–	–	–	–	–	–	–	–	–	67.68	3.101	0.622	67.68
Medial lemniscus L	–	–	–	–	–	–	–	2.72	3.121	0.586	68.10	3.099	0.634	65.38
Inferior cerebellar peduncle R	–	–	–	–	–	–	–	–	–	–	10.23	2.744	0.547	10.23
Inferior cerebellar peduncle L	–	–	–	–	–	–	–	1.14	2.660	0.571	8.57	2.683	0.583	7.44
Superior cerebellar peduncle R	–	–	–	5.44	4.544	0.745	5.44	–	–	–	26.31	3.414	0.597	26.31
Superior cerebellar peduncle L	–	–	–	–	–	–	–	–	–	–	23.29	2.995	0.587	23.29
Cerebral peduncle R	–	–	–	–	–	–	–	0.18	1.799	0.427	14.05	2.890	0.592	13.87
Cerebral peduncle L	–	–	–	–	–	–	–	8.08	2.868	0.573	30.42	3.221	0.598	22.34
Anterior limb of internal capsule R	11.66	2.712	0.540	–	–	–	−11.66	26.77	2.189	0.477	13.77	3.052	0.534	−13.00
Anterior limb of internal capsule L	3.35	2.338	0.300	–	–	–	−3.35	28.93	1.964	0.422	18.82	3.103	0.438	−10.11
Posterior limb of internal capsule R	–	–	–	2.64	3.397	0.658	2.64	6.98	1.815	0.408	10.97	3.035	0.505	4.00
Posterior limb of internal capsule L	–	–	–	–	–	–	–	2.45	1.952	0.433	5.60	2.869	0.492	3.14
Retrolenticular part of internal capsule R	5.49	2.985	0.448	–	–	–	−5.49	13.40	2.032	0.383	2.35	2.849	0.589	−11.05
Retrolenticular part of internal capsule L	8.79	3.331	0.575	–	–	–	−8.79	22.68	2.113	0.440	0.16	2.584	0.586	−22.52
Anterior corona radiata R	36.78	2.588	0.473	8.99	3.471	0.659	−27.79	40.44	2.303	0.443	0.10	2.661	0.365	−40.34
Anterior corona radiata L	30.01	2.999	0.517	–	–	–	−30.01	32.79	2.698	0.499	4.31	2.775	0.376	−28.49
Superior corona radiata R	38.95	2.984	0.450	–	–	–	−38.95	27.36	2.237	0.423	–	–	–	−27.36
Superior corona radiata L	34.12	2.636	0.385	–	–	–	−34.12	32.78	2.152	0.387	–	–	–	−32.78
Posterior corona radiata R	12.20	2.493	0.423	–	–	–	−12.20	5.28	1.884	0.357	–	–	–	−5.28
Posterior corona radiata L	3.39	3.243	0.578	–	–	–	−3.39	6.52	2.374	0.478	–	–	–	−6.52
Posterior thalamic radiation R	4.41	3.137	0.537	–	–	–	−4.41	25.33	2.144	0.406	–	–	–	−25.33
Posterior thalamic radiation L	8.62	3.395	0.573	0.40	4.048	0.706	−8.22	18.07	2.497	0.472	–	–	–	−18.07
Sagittal stratum R	20.78	3.097	0.586	–	–	–	−20.78	31.15	2.410	0.498	0.85	3.554	0.718	−30.30
Sagittal stratum L	3.72	3.719	0.684	–	–	–	−3.72	26.45	2.490	0.518	–	–	–	−26.45
External capsule R	26.52	2.683	0.457	0.57	3.900	0.725	−25.95	26.91	2.231	0.456	5.93	3.355	0.482	−20.98
External capsule L	0.55	3.002	0.551	–	–	–	−0.55	22.59	2.201	0.444	0.45	2.664	0.357	−22.14
Cingulum (cingulate gyrus) R	9.74	2.406	0.523	–	–	–	−9.74	12.34	2.078	0.449	–	–	–	−12.34
Cingulum (cingulate gyrus) L	34.10	2.781	0.477	–	–	–	−34.10	22.79	2.241	0.460	–	–	–	−22.79
Fornix (cres)/Stria terminalis R	–	–	–	–	–	–	–	12.63	2.211	0.416	0.18	3.932	0.637	−12.46
Fornix (cres)/Stria terminalis L	–	–	–	–	–	–	–	10.31	1.897	0.411	13.07	3.158	0.650	2.76
Superior longitudinal fasciculus R	19.98	2.698	0.459	0.09	3.670	0.521	−19.89	12.37	1.975	0.422	–	–	–	−12.37
Superior longitudinal fasciculus L	6.46	2.748	0.492	–	–	–	−6.46	12.90	2.301	0.460	–	–	–	−12.90
Superior fronto-occipital fasciculus R	31.36	2.417	0.479	–	–	–	−31.36	46.94	2.067	0.457	–	–	–	−46.94
Superior fronto-occipital fasciculus L	27.22	2.717	0.422	–	–	–	−27.22	34.52	2.111	0.411	–	–	–	−34.52
Uncinate fasciculus R	38.16	3.425	0.609	–	–	–	−38.16	72.63	2.418	0.500	–	–	–	−72.63
Uncinate fasciculus L	–	–	–	–	–	–	–	6.12	1.844	0.395	–	–	–	−6.12
Tapetum R	10.07	3.438	0.628	–	–	–	−10.07	11.74	2.318	0.465	–	–	–	−11.74
Tapetum L	12.17	3.828	0.631	–	–	–	−12.17	8.33	2.870	0.508	–	–	–	−8.33

Results are expressed as *t*-values and Spearman’s rank correlation coefficient (*ρ*). The percentage (%) indicates the cluster volume within the respective white matter region based on the atlas. The column ‘Δ vol% PD (ON) - PD (OFF)’ represents the difference in the percentage of significant cluster volumes between the ON and OFF states.

*t*, *t*-value; *g*, Hedges’ *g* (effect-size); vol, volume; FWF, Free Water Fraction; ODI, Orientation Dispersion Index; NDI, Neurite Density Index; HC, healthy control; PD, Parkinson’s disease.

### 3.6 NODDI metrics and correlations with MDS-UPDRS III/H&Y scores

**Figure 5:**
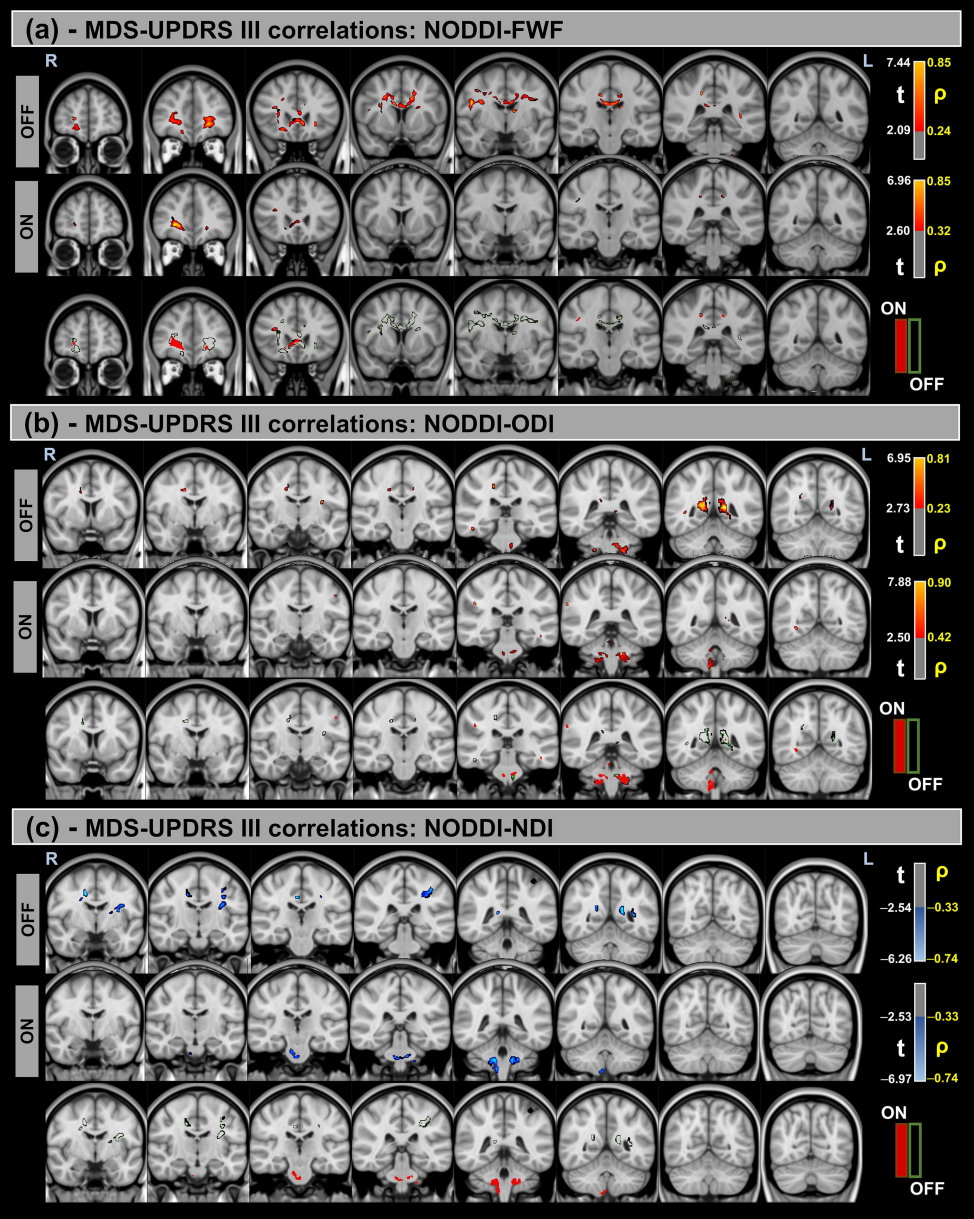
Voxel-based correlations between NODDI metrics and MDS-UPDRS-III scores in the Parkinson’s Disease (PD) group. **(a,b)** Positive correlations with the FWF and ODI are shown warm colors (red-yellow). **(c)** Negative correlations with the NDI are shown cool colors (blue-light blue). The color bars represent *t*-values and corresponding correlation coefficients (*ρ*). Differences between OFF and ON states (green for OFF and red for ON) indicate a reduction in correlation strength during the ON state.

**Figure 6:**
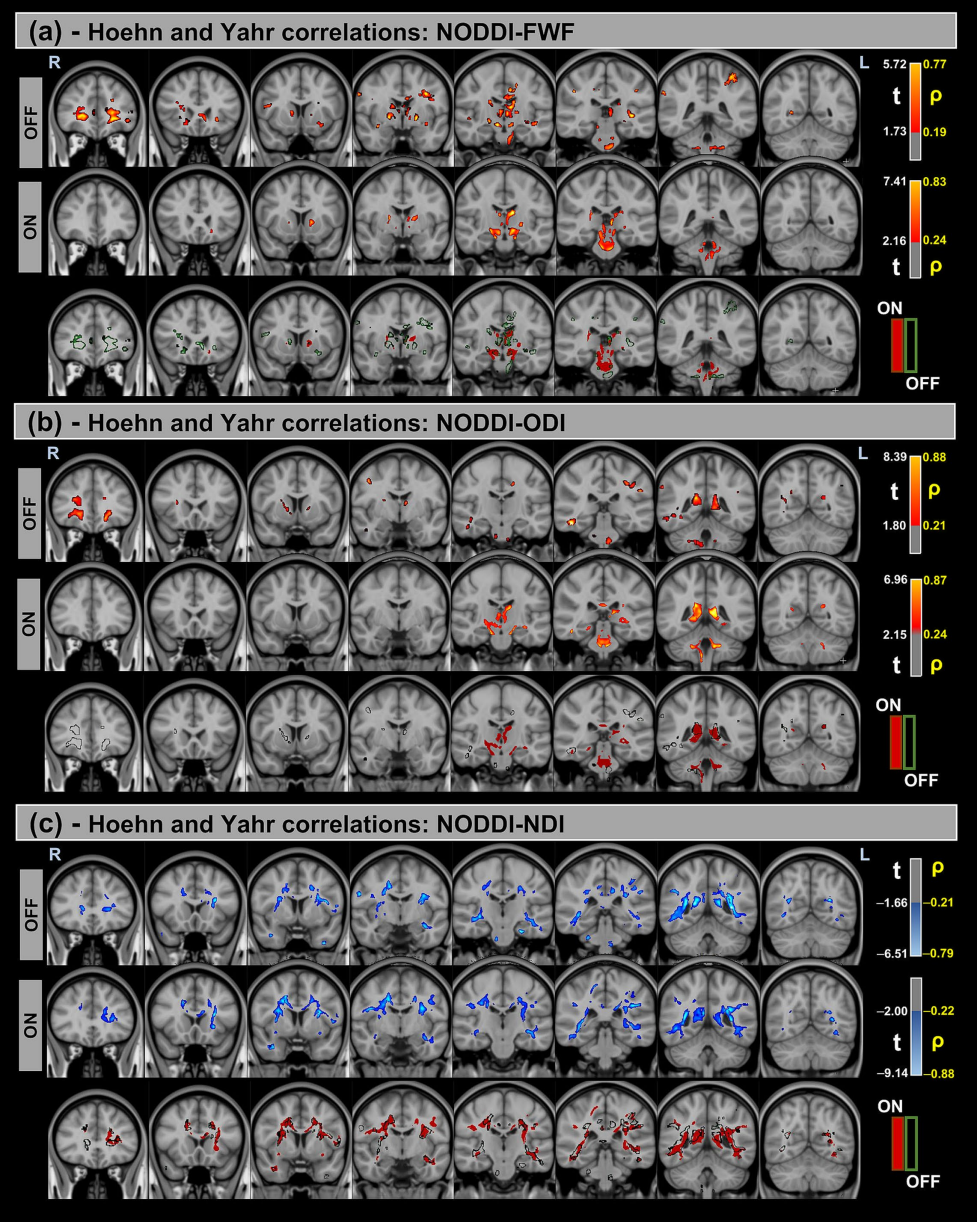
Voxel-based correlations between NODDI metrics and H&Y scores in the Parkinson’s Disease (PD) group. **(a,b)** Positive correlations with the FWF and ODI are shown warm colors (red-yellow). **(c)** Negative correlations with the NDI are shown cool colors (blue-light blue). The color bars represent *t*-values and corresponding correlation coefficients (*ρ*). Differences between OFF and ON states (green for OFF and red for ON) indicate a reduction in correlation strength during the ON state.

Figures 5, 6 show voxel-based correlations between NODDI metrics and MDS-UPDRS III/H&Y in the PD group across OFF and ON states.

Significant positive correlations were observed between MDS-UPDRS-III and both FWF and ODI metrics, whereas significant negative correlations were found for the NDI. Additionally, differences in correlation patterns were detected between the OFF and ON states.

Similar trends were observed for the H&Y scores, with significant correlations in the same direction of MDS-UPDRS-III for all NODDI metrics.

Table 7 provides a summary of these findings, including cluster volumes, *t*-values, *ρ*-values, and the percentage change in volume between the OFF and ON states.

**Table 7:** Voxel-based correlations between NODDI metrics and clinical scores (MDS-UPDRS III and Hoehn and Yahr) in PD groups during OFF and ON states.

Atlas	MDS-UPDRS III							Hoehn and Yahr						
	FWF - PD (OFF)			FWF - PD (ON)			Δ vol% PD (ON)- PD (OFF)	FWF - PD (OFF)			FWF - PD (ON)			Δ vol% PD (ON) – PD (OFFf)
	vol (%)	*t*	*ρ*	vol (%)	*t*	*ρ*	Δvol (%)	vol (%)	*t*	*ρ*	vol (%)	*t*	*ρ*	Δvol (%)
JHU Atlas														
Anterior Thalamic Radiation L	4.28	3.044	0.527	0.67	3.360	0.607	−3.61	15.36	2.300	0.444	19.88	2.938	0.548	4.51
Anterior Thalamic Radiation R	7.97	2.615	0.511	4.32	3.812	0.621	−3.65	14.02	2.278	0.446	12.44	2.802	0.555	−1.58
Cortical spinal tract L	1.12	3.270	0.628	–	–	–	−1.12	5.05	2.289	0.489	11.33	2.807	0.532	6.29
Cortical spinal tract R	–	–	–	–	–	–	–	3.06	2.353	0.476	13.31	2.869	0.563	10.25
Cingulum cingulate gyrus L	3.64	2.631	0.509	0.05	4.301	0.657	−3.59	5.02	2.311	0.451	–	–	–	−5.02
Cingulum cingulate gyrus R	1.19	2.608	0.584	0.26	4.505	0.666	−0.93	1.02	2.413	0.477	–	–	–	−1.02
Forceps Major	1.17	4.219	0.651	0.04	4.262	0.683	−1.13	3.38	2.618	0.483	0.94	4.715	0.622	−2.44
Forceps Minor	7.85	2.694	0.557	3.01	3.299	0.575	−4.83	9.29	2.241	0.433	–	–	–	−9.29
Inferior fronto-occipital fasc L	5.37	3.037	0.535	1.19	3.663	0.630	−4.18	8.98	2.399	0.446	0.57	2.518	0.375	−8.42
Inferior fronto-occipital fasc R	8.41	2.678	0.519	5.40	3.765	0.624	−3.02	9.84	2.298	0.440	0.03	2.558	0.649	−9.81
Inferior Longitudinal fasc L	0.31	3.876	0.683	0.61	4.193	0.630	0.30	1.42	2.297	0.441	–	–	–	−1.42
Inferior Longitudinal fasc R	0.30	4.232	0.729	–	4.977	0.801	−0.29	1.22	2.658	0.491	–	–	–	−1.22
Superior Longitudinal fasc L	0.93	2.564	0.504	0.06	4.602	0.582	−0.87	1.61	2.516	0.512	–	–	–	−1.61
Superior Longitudinal fasc R	3.88	2.793	0.545	0.29	3.814	0.550	−3.58	0.90	2.607	0.507	–	–	–	−0.90
Uncinate fasc L	10.70	2.941	0.517	1.75	3.339	0.597	−8.95	14.77	2.366	0.439	1.52	2.541	0.372	−13.25
Uncinate fasc R	10.61	2.586	0.519	9.90	3.734	0.602	−0.70	16.53	2.320	0.451	–	–	–	−16.53
Sup Longitudinal fasc temporal L	0.95	2.592	0.501	–	–	–	−0.95	1.14	2.447	0.504	–	–	–	−1.14
Sup Longitudinal fasc temporal R	4.61	2.807	0.542	0.14	3.716	0.508	−4.46	0.39	2.379	0.458	–	–	–	−0.39
ICBM81 Atlas														
Middle cerebellar peduncle	–	–	–	–	–	–	–	4.14	2.262	0.487	1.90	2.432	0.449	−2.24
Pontine crossing tract (a part of MCP)	2.00	3.324	0.642	–	–	–	−2.00	1.13	2.178	0.464	91.00	2.803	0.573	89.87
Genu of corpus callosum	14.94	2.646	0.567	5.17	3.098	0.566	−9.76	8.11	2.089	0.441	–	–	–	−8.11
Body of corpus callosum	36.45	2.837	0.556	0.29	4.232	0.627	−36.15	7.77	2.338	0.500	–	–	–	−7.77
Splenium of corpus callosum	1.60	2.334	0.508	–	–	–	−1.60	0.98	2.209	0.437	2.29	4.692	0.623	1.31
Fornix (column and body of fornix)	–	–	–	–	–	–	–	19.27	2.005	0.416	–	–	–	−19.27
Corticospinal tract R	–	–	–	–	–	–	–	7.86	2.166	0.480	10.72	2.762	0.558	2.86
Corticospinal tract L	3.28	3.430	0.691	–	–	–	−3.28	26.72	2.227	0.484	10.00	2.608	0.568	−16.72
Medial lemniscus R	–	–	–	–	–	–	–	–	–	–	83.62	2.757	0.570	83.62
Medial lemniscus L	–	–	–	–	–	–	–	8.87	2.507	0.495	72.10	2.854	0.588	63.23
Inferior cerebellar peduncle R	–	–	–	–	–	–	–	0.10	2.178	0.489	9.30	2.641	0.527	9.19
Inferior cerebellar peduncle L	–	–	–	–	–	–	–	7.23	2.058	0.469	19.83	2.498	0.512	12.60
Superior cerebellar peduncle R	–	–	–	–	–	–	–	–	–	–	34.17	2.971	0.572	34.17
Superior cerebellar peduncle L	–	–	–	–	–	–	–	–	–	–	27.72	2.840	0.532	27.72
Cerebral peduncle R	–	–	–	–	–	–	–	11.06	2.927	0.511	58.91	2.864	0.542	47.85
Cerebral peduncle L	–	–	–	–	–	–	–	7.33	2.420	0.483	56.85	2.925	0.543	49.52
Anterior limb of internal capsule R	2.33	2.770	0.557	–	–	–	−2.33	10.29	2.149	0.473	3.66	3.501	0.570	−6.63
Anterior limb of internal capsule L	0.30	2.345	0.466	–	–	–	−0.30	8.08	1.991	0.433	26.81	2.811	0.469	18.72
Posterior limb of internal capsule R	–	–	–	–	–	–	–	9.03	2.224	0.481	9.03	2.791	0.506	–
Posterior limb of internal capsule L	–	–	–	–	–	–	–	3.52	2.381	0.495	15.88	2.720	0.436	12.37
Retrolenticular part of internal capsule R	–	–	–	–	–	–	–	–	–	–	4.10	2.517	0.566	4.10
Retrolenticular part of internal capsule L	3.12	4.108	0.684	–	–	–	−3.12	8.55	2.556	0.481	1.58	2.670	0.491	−6.97
Anterior corona radiata R	22.73	2.564	0.479	13.99	3.809	0.601	−8.75	20.60	2.280	0.410	–	–	–	−20.60
Anterior corona radiata L	18.49	2.964	0.515	0.96	3.278	0.567	−17.53	19.96	2.552	0.442	0.76	2.597	0.312	−19.21
Superior corona radiata R	17.95	2.793	0.433	–	–	–	−17.95	2.60	2.239	0.469	0.03	2.273	0.605	−2.57
Superior corona radiata L	11.31	2.430	0.402	–	–	–	−11.31	5.69	2.137	0.398	–	–	–	−5.69
Posterior corona radiata R	1.69	4.407	0.679	0.59	4.121	0.530	−1.10	0.89	2.699	0.474	0.35	2.723	0.660	−0.54
Posterior corona radiata L	–	–	–	–	–	–	–	–	–	–	0.03	2.377	0.577	0.03
Posterior thalamic radiation L	1.08	3.894	0.704	0.73	3.999	0.650	−0.35	–	–	–	–	–	–	–
Sagittal stratum L	–	–	–	–	–	–	–	2.06	1.863	0.363	–	–	–	−2.06
External capsule R	4.87	2.694	0.506	–	–	–	−4.87	8.63	2.325	0.476	–	–	–	−8.63
External capsule L	0.30	2.472	0.573	–	–	–	−0.30	9.84	2.030	0.408	2.13	2.517	0.387	−7.71
Cingulum (cingulate gyrus) R	0.34	2.276	0.564	0.34	4.453	0.659	–	1.37	2.679	0.510	–	–	–	−1.37
Cingulum (cingulate gyrus) L	3.89	2.631	0.547	–	–	–	−3.89	3.24	2.577	0.517	–	–	–	−3.24
Fornix (cres)/Stria terminalis R	–	–	–	–	–	–	–	3.20	2.635	0.455	–	–	–	−3.20
Fornix (cres)/Stria terminalis L	–	–	–	–	–	–	–	12.62	2.102	0.398	21.87	3.014	0.634	9.24
Superior longitudinal fasciculus R	6.33	2.796	0.533	0.36	3.711	0.515	−5.96	0.03	2.214	0.501	–	–	–	−0.03
Superior longitudinal fasciculus L	1.71	2.437	0.499	–	–	–	−1.71	1.85	2.327	0.493	–	–	–	−1.85
Superior fronto-occipital fasciculus R	–	–	–	–	–	–	–	8.48	2.389	0.513	–	–	–	−8.48
Superior fronto-occipital fasciculus L	12.62	2.526	0.473	–	–	–	−12.62	1.38	2.068	0.394	–	–	–	−1.38
Uncinate fasciculus R	–	–	–	–	–	–	–	17.89	2.627	0.536	–	–	–	−17.89
Uncinate fasciculus L	–	–	–	–	–	–	–	1.60	1.839	0.406	–	–	–	−1.60

Atlas	MDS-UPDRS III							Hoehn and Yahr						
	ODI - PD (OFF)			ODI - PD (ON)			Δ vol% PD (ON)- PD (OFF)	ODI - PD (OFF)			ODI - PD (ON)			Δ vol% PD (ON) – PD (OFFf)
	vol (%)	*t*	*ρ*	vol (%)	*t*	*ρ*	Δvol (%)	vol (%)	*t*	*ρ*	vol (%)	*t*	*ρ*	Δvol (%)
JHU Atlas														
Anterior Thalamic Radiation L	0.24	3.198	0.639	–	–	0.642	−0.24	4.17	2.532	0.504	10.86	2.951	0.592	6.69
Anterior Thalamic Radiation R	0.20	3.501	0.687	0.94	2.851	0.629	0.73	5.01	2.411	0.503	8.12	2.850	0.565	3.11
Cortical spinal tract L	0.57	3.026	0.631	0.74	2.950	0.658	0.17	2.11	2.465	0.523	5.78	3.255	0.605	3.67
Cortical spinal tract R	0.02	2.995	0.638	1.35	2.754	0.618	1.33	0.96	2.301	0.505	11.29	2.937	0.581	10.33
Cingulum cingulate gyrus L	0.55	3.131	0.628	–	–	–	−0.55	3.24	2.972	0.552	–	–	–	−3.24
Cingulum cingulate gyrus R	0.70	3.107	0.431	–	–	–	−0.70	0.22	2.369	0.489	–	–	–	−0.22
Cingulum Hippo L	4.80	3.396	0.576	–	–	–	−4.80	1.18	2.510	0.531	10.83	2.887	0.605	9.65
Cingulum Hippo R	1.93	3.646	0.648	–	–	–	−1.93	1.61	2.649	0.549	25.22	2.867	0.623	23.61
Forceps Major	7.48	3.855	0.639	0.34	4.386	0.744	−7.14	11.39	2.630	0.510	13.64	3.640	0.614	2.25
Forceps Minor	0.11	3.597	0.712	–	–	–	−0.11	5.15	2.497	0.494	–	–	–	−5.15
Inferior fronto-occipital fasc L	–	–	–	0.11	4.547	0.727	0.11	3.34	2.565	0.519	0.42	2.712	0.562	−2.91
Inferior fronto-occipital fasc R	0.43	3.752	0.683	0.37	4.115	0.694	−0.05	10.19	2.377	0.474	–	–	–	−10.19
Inferior Longitudinal fasc L	–	–	–	0.30	4.370	0.680	0.30	–	–	0.567	–	–	–	–
Inferior Longitudinal fasc R	0.50	4.063	0.651	0.50	4.038	0.679	–	10.66	2.543	0.483	0.43	3.712	0.647	−10.23
Superior Longitudinal fasc L	0.13	4.276	0.638	0.18	3.937	0.642	0.05	2.62	2.673	0.534	–	–	–	−2.62
Superior Longitudinal fasc R	0.17	3.957	0.597	0.17	4.255	0.606	−0.01	2.88	2.699	0.503	–	–	–	−2.88
Uncinate fasc L	–	–	–	–	–	–	–	7.88	2.586	0.515	–	–	–	−7.88
Uncinate fasc R	0.52	3.516	0.706	–	–	–	−0.52	6.52	2.497	0.519	–	–	–	−6.52
Sup Longitudinal fasc temporal L	0.02	4.044	0.669	0.11	4.299	0.628	0.10	0.81	2.636	0.508	–	–	–	−0.81
Sup Longitudinal fasc temporal R	0.58	3.957	0.598	0.35	3.900	0.573	−0.23	7.91	2.668	0.494	–	–	–	−7.91
ICBM81 Atlas														
Middle cerebellar peduncle	0.58	3.303	0.613	2.80	3.100	0.634	2.22	4.27	2.322	0.502	7.51	3.238	0.501	3.24
Pontine crossing tract (a part of MCP)	0.20	2.940	0.650	1.87	2.877	0.665	1.67	0.80	2.194	0.489	77.33	3.080	0.625	76.53
Genu of corpus callosum	–	–	–	–	–	–	–	0.18	2.191	0.470	–	–	–	−0.18
Body of corpus callosum	1.82	3.291	0.558	–	–	–	−1.82	0.14	3.041	0.488	0.32	2.704	0.625	0.18
Splenium of corpus callosum	18.34	3.797	0.639	0.31	4.121	0.729	−18.04	20.33	2.727	0.540	36.74	3.511	0.607	16.40
Corticospinal tract R	–	–	–	10.79	2.718	0.612	10.79	2.06	2.150	0.489	2.72	2.932	0.583	0.66
Corticospinal tract L	0.95	2.966	0.626	3.21	3.011	0.676	2.26	7.15	2.404	0.516	4.31	3.149	0.582	−2.85
Medial lemniscus R	–	–	–	4.06	2.974	0.636	4.06	–	–	–	77.97	3.304	0.621	77.97
Medial lemniscus L	2.72	3.052	0.618	–	–	–	−2.72	12.30	2.167	0.486	69.96	3.273	0.626	57.65
Inferior cerebellar peduncle R	12.19	3.325	0.585	21.18	3.781	0.690	8.99	10.85	2.248	0.480	31.30	3.448	0.590	20.45
Inferior cerebellar peduncle L	12.91	3.284	0.637	9.40	3.170	0.672	−3.51	3.00	2.115	0.455	27.69	3.564	0.660	24.69
Superior cerebellar peduncle R	–	–	–	6.35	2.663	0.626	6.35	–	–	–	14.82	2.954	0.513	14.82
Superior cerebellar peduncle L	–	–	–	–	–	–	–	–	–	–	26.61	2.880	0.554	26.61
Cerebral peduncle R	–	–	–	–	–	–	–	–	–	–	42.98	2.784	0.572	42.98
Cerebral peduncle L	–	–	–	–	–	–	–	–	–	–	13.48	3.186	0.649	13.48
Anterior limb of internal capsule R	–	–	–	–	–	–	–	9.11	2.633	0.515	–	–	–	−9.11
Anterior limb of internal capsule L	–	–	–	–	–	–	–	3.48	2.705	0.527	–	–	–	−3.48
Posterior limb of internal capsule R	–	–	–	–	–	–	–	0.24	2.559	0.446	4.05	2.798	0.612	3.81
Posterior limb of internal capsule L	–	–	–	–	–	–	–	–	–	–	0.45	3.472	0.598	0.45
Retrolenticular part of internal capsule R	–	–	–	–	–	–	–	–	–	–	3.58	2.717	0.545	3.58
Retrolenticular part of internal capsule L	–	–	–	–	–	–	–	–	–	–	1.66	3.325	0.574	1.66
Anterior corona radiata R	1.14	3.499	0.672	–	–	–	−1.14	25.52	2.432	0.498	–	–	–	−25.52
Anterior corona radiata L	–	–	–	–	–	–	–	10.30	2.661	0.536	–	–	–	−10.30
Superior corona radiata R	3.96	3.392	0.651	–	–	–	−3.96	–	–	–	–	–	–	–
Superior corona radiata L	0.47	4.503	0.656	–	–	–	−0.47	0.59	2.868	0.451	0.37	3.731	0.599	−0.21
Posterior corona radiata R	0.32	3.025	0.603	–	–	–	−0.32	0.59	2.508	0.519	–	–	–	−0.59
Posterior corona radiata L	–	–	–	–	–	–	–	0.22	2.302	0.401	1.78	3.834	0.590	1.56
Posterior thalamic radiation R	0.15	3.784	0.643	0.63	3.872	0.704	0.48	16.31	2.240	0.446	–	–	–	−16.31
Sagittal stratum R	–	–	–	0.63	3.894	0.669	0.63	18.22	2.316	0.454	0.72	3.514	0.631	−17.50
Sagittal stratum L	–	–	–	–	–	–	–	–	–	–	3.05	2.567	0.539	3.05
External capsule L	0.14	4.322	0.577	–	–	–	−0.14	0.07	2.186	0.482	0.39	3.601	0.548	0.32
Cingulum (cingulate gyrus) R	2.01	3.023	0.583	–	–	–	−2.01	2.56	2.354	0.512	2.60	2.691	0.608	0.04
Cingulum (cingulate gyrus) L	2.29	3.247	0.619	–	–	–	−2.29	0.91	2.265	0.468	0.36	2.342	0.510	−0.55
Cingulum (hippocampus) R	7.04	3.109	0.596	–	–	–	−7.04	0.08	1.917	0.443	33.33	2.877	0.640	33.25
Cingulum (hippocampus) L	3.64	3.195	0.612	–	–	–	−3.64	2.25	2.581	0.534	7.45	2.824	0.603	5.19
Fornix (cres)/Stria terminalis L	–	–	–	–	–	–	–	–	–	–	44.53	2.845	0.594	44.53
Superior longitudinal fasciculus R	0.45	3.970	0.589	0.45	3.934	0.568	–	3.19	2.688	0.493	–	–	–	−3.19
Superior longitudinal fasciculus L	0.09	4.273	0.706	–	–	–	−0.09	2.03	2.511	0.494	–	–	–	−2.03

Atlas	MDS-UPDRS III							Hoehn and Yahr						
	NDI - PD (OFF)			NDI - PD (ON)			Δ vol% PD (ON)- PD (OFF)	NDI - PD (OFF)			NDI - PD (ON)			Δ vol% PD (ON) – PD (OFFf)
	vol (%)	*t*	*ρ*	vol (%)	*t*	*ρ*	Δvol (%)	vol (%)	*t*	*ρ*	vol (%)	*t*	*ρ*	Δvol (%)
JHU Atlas														
Anterior Thalamic Radiation L	–	–	–	–	–	–	–	5.86	−2.274	−0.444	6.22	−2.688	−0.346	0.37
Anterior Thalamic Radiation R	–	–	–	0.25	−3.158	−0.642	0.25	0.62	−2.042	−0.420	0.96	−2.465	−0.266	0.34
Cortical spinal tract L	0.39	−2.776	−0.509	0.86	−2.975	−0.652	0.47	6.53	−2.219	−0.474	9.52	−2.621	−0.378	2.99
Cortical spinal tract R	–	–	–	2.81	−2.965	−0.619	2.81	4.52	−2.115	−0.449	9.76	−2.549	−0.328	5.24
Cingulum cingulate gyrus L	–	–	–	–	–	–	–	12.08	−2.280	−0.414	2.41	−2.366	−0.388	−9.67
Cingulum cingulate gyrus R	0.64	−4.260	−0.487	–	–	–	−0.64	5.17	−2.308	−0.408	7.08	−2.337	−0.232	1.91
Cingulum Hippo L	0.41	−3.675	−0.476	–	–	–	−0.41	8.26	−3.014	−0.545	6.54	−2.589	−0.340	−1.72
Cingulum Hippo R	–	–	–	–	–	–	–	1.20	−2.092	−0.444	6.83	−2.325	−0.334	5.63
Forceps Major	1.63	−3.667	−0.493	–	–	–	−1.63	13.53	−2.520	−0.468	15.00	−2.884	−0.431	1.48
Forceps Minor	–	–	–	–	–	–	–	2.25	−2.197	−0.447	4.76	−2.430	−0.368	2.51
Inferior fronto-occipital fasc L	0.46	−3.340	−0.606	–	–	–	−0.46	15.20	−2.394	−0.458	20.49	−2.849	−0.353	5.29
Inferior fronto-occipital fasc R	–	–	–	–	–	–	–	16.69	−2.377	−0.461	12.13	−2.838	−0.361	−4.56
Inferior Longitudinal fasc L	0.39	−3.383	−0.622	–	–	–	−0.39	13.79	−2.364	−0.467	21.17	−2.762	−0.356	7.38
Inferior Longitudinal fasc R	–	–	–	–	–	–	–	15.74	−2.212	−0.440	11.97	−2.609	−0.331	−3.77
Superior Longitudinal fasc L	4.46	−3.057	−0.458	–	–	–	−4.46	12.96	−2.289	−0.456	18.74	−2.740	−0.347	5.77
Superior Longitudinal fasc R	–	–	–	–	–	–	–	9.15	−2.202	−0.434	12.42	−2.666	−0.390	3.28
Uncinate fasc L	0.10	−3.028	−0.441	–	–	–	−0.10	9.16	−2.306	−0.429	11.52	−2.676	−0.297	2.36
Uncinate fasc R	–	–	–	–	–	–	–	0.17	−2.618	−0.548	3.64	−2.613	−0.349	3.47
Sup Longitudinal fasc temporal L	4.15	−3.048	−0.442	–	–	–	−4.15	16.38	−2.219	−0.442	24.70	−2.733	−0.336	8.32
Sup Longitudinal fasc temporal R	–	–	–	–	–	–	–	16.96	−2.128	−0.434	14.26	−2.578	−0.397	−2.70
ICBM81 Atlas														
Middle cerebellar peduncle	–	–	–	3.78	−3.123	−0.615	3.78	1.43	−2.631	−0.546	–	–	–	−1.43
Pontine crossing tract (a part of MCP)	–	–	–	2.47	−3.195	−0.653	2.47	0.27	−2.497	−0.547	–	–	–	−0.27
Genu of corpus callosum	–	–	–	–	–	–	–	8.12	−2.207	−0.450	11.31	−2.534	−0.382	3.19
Body of corpus callosum	1.39	−3.693	−0.420	–	–	–	−1.39	6.86	−2.312	−0.421	16.43	−2.766	−0.345	9.58
Splenium of corpus callosum	5.07	−3.667	−0.491	–	–	–	−5.07	25.48	−2.486	−0.445	33.42	−2.808	−0.384	7.94
Corticospinal tract R	–	–	–	23.42	−2.948	−0.613	23.42	0.15	−2.345	−0.504	–	–	–	−0.15
Corticospinal tract L	–	–	–	0.88	−2.680	−0.645	0.88	4.45	−2.573	−0.541	–	–	–	−4.45
Medial lemniscus R	–	–	–	7.68	−3.252	−0.647	7.68	–	–	–	–	–	–	–
Medial lemniscus L	–	–	–	1.43	−2.702	−0.629	1.43	1.29	−2.399	−0.525	–	–	–	−1.29
Inferior cerebellar peduncle R	–	–	–	13.22	−3.488	−0.666	13.22	–	–	–	–	–	–	–
Inferior cerebellar peduncle L	–	–	–	5.17	−2.937	−0.642	5.17	–	–	–	–	–	–	–
Anterior limb of internal capsule R	–	–	–	–	–	–	–	1.69	−1.916	−0.385	5.74	−2.688	−0.333	4.05
Anterior limb of internal capsule L	1.46	−2.946	−0.326	–	–	–	−1.46	3.84	−1.921	−0.363	4.08	−2.379	−0.189	0.23
Posterior limb of internal capsule R	–	–	–	–	–	–	–	1.23	−1.786	−0.388	4.98	−2.395	−0.251	3.76
Posterior limb of internal capsule L	0.80	−3.092	−0.296	–	–	–	−0.80	1.81	−1.893	−0.333	5.41	−2.375	−0.165	3.60
Retrolenticular part of internal capsule R	–	–	–	–	–	–	–	34.16	−2.364	−0.430	39.76	−3.116	−0.347	5.61
Retrolenticular part of internal capsule L	–	–	–	–	–	–	–	13.69	−2.274	−0.407	22.96	−2.768	−0.248	9.28
Anterior corona radiata R	–	–	–	–	–	–	–	6.21	−2.169	−0.439	10.53	−2.624	−0.336	4.32
Anterior corona radiata L	1.58	−3.071	−0.445	–	–	–	−1.58	22.64	−2.166	−0.417	20.80	−2.506	−0.355	−1.84
Superior corona radiata R	3.09	−3.661	−0.466	–	–	–	−3.09	27.56	−2.234	−0.430	56.44	−2.894	−0.337	28.88
Superior corona radiata L	3.77	−2.994	−0.401	–	–	–	−3.77	19.81	−2.120	−0.428	35.44	−2.837	−0.407	15.64
Posterior corona radiata R	–	–	–	–	–	–	–	35.43	−2.302	−0.468	33.23	−2.715	−0.393	−2.20
Posterior corona radiata L	0.24	−2.904	−0.592	–	–	–	−0.24	36.59	−2.453	−0.506	36.19	−2.744	−0.426	−0.40
Posterior thalamic radiation R	–	–	–	–	–	–	–	50.13	−2.461	−0.494	31.32	−2.930	−0.409	−18.81
Posterior thalamic radiation L	3.04	−3.365	−0.624	–	–	–	−3.04	32.88	−2.678	−0.530	30.89	−2.857	−0.434	−1.99
Sagittal stratum R	–	–	–	–	–	–	–	57.27	−2.184	−0.430	47.62	−2.580	−0.287	−9.65
Sagittal stratum L	–	–	–	–	–	–	–	36.89	−2.401	−0.463	36.40	−3.202	−0.374	−0.49
External capsule R	–	–	–	–	–	–	–	24.42	−2.041	−0.403	17.95	−2.506	−0.284	−6.47
External capsule L	3.99	−3.033	−0.363	–	–	–	−3.99	10.01	−2.141	−0.395	19.71	−2.795	−0.274	9.70
Cingulum (cingulate gyrus) R	1.45	−4.255	−0.465	–	–	–	−1.45	6.15	−2.306	−0.391	8.41	−2.500	−0.293	2.26
Cingulum (cingulate gyrus) L	–	–	–	–	–	–	–	12.21	−2.085	−0.394	2.11	−2.365	−0.314	−10.11
Cingulum (hippocampus) R	–	–	–	–	–	–	–	–	–	–	5.18	−2.315	−0.345	5.18
Cingulum (hippocampus) L	–	–	–	–	–	–	–	10.91	−3.264	−0.562	3.90	−2.210	−0.302	−7.01
Fornix (cres)/Stria terminalis R	–	–	–	–	–	–	–	4.89	−2.001	−0.413	14.50	−2.502	−0.339	9.61
Fornix (cres)/Stria terminalis L	–	–	–	–	–	–	–	15.91	−2.316	−0.449	53.78	−2.835	−0.301	37.87
Superior longitudinal fasciculus R	–	–	–	–	–	–	–	9.19	−2.096	−0.405	17.75	−2.675	−0.397	8.57
Superior longitudinal fasciculus L	10.20	−3.089	−0.445	–	–	–	−10.20	20.36	−2.167	−0.434	36.56	−2.756	−0.332	16.20
Superior fronto-occipital fasciculus R	–	–	–	–	–	–	–	22.88	−1.898	−0.405	34.91	−2.443	−0.198	12.03
Superior fronto-occipital fasciculus L	–	–	–	–	–	–	–	13.41	−1.930	−0.431	1.78	−2.176	−0.460	−11.64
Uncinate fasciculus R	–	–	–	–	–	–	–	–	–	–	20.79	−2.236	−0.298	20.79
Uncinate fasciculus L	–	–	–	–	–	–	–	31.91	−2.119	−0.441	28.99	−2.961	−0.479	−2.93
Tapetum R	–	–	–	–	–	–	–	60.40	−2.804	−0.556	72.99	−3.262	−0.520	12.58
Tapetum L	4.00	−3.247	−0.660	–	–	–	−4.00	50.33	−2.511	−0.540	57.50	−3.253	−0.597	7.17

Results are expressed as *t*-values and Spearman’s rank correlation coefficient (*ρ*). The percentage (%) indicates the cluster volume within the respective white matter region based on the atlas. The column ‘Δ vol% PD (on) - PD (off)’ represents the difference in the percentage of significant cluster volumes between the ON and OFF states.

*t*, *t*-value; *g*, Hedges’ *g* (effect-size); vol, volume; FWF, Free Water Fraction; ODI, Orientation Dispersion Index; NDI, Neurite Density Index; HC, healthy control; PD, Parkinson’s disease.

### 3.7 Bootstrap distribution of AUCs and differences in AUC

**Figure 7:**
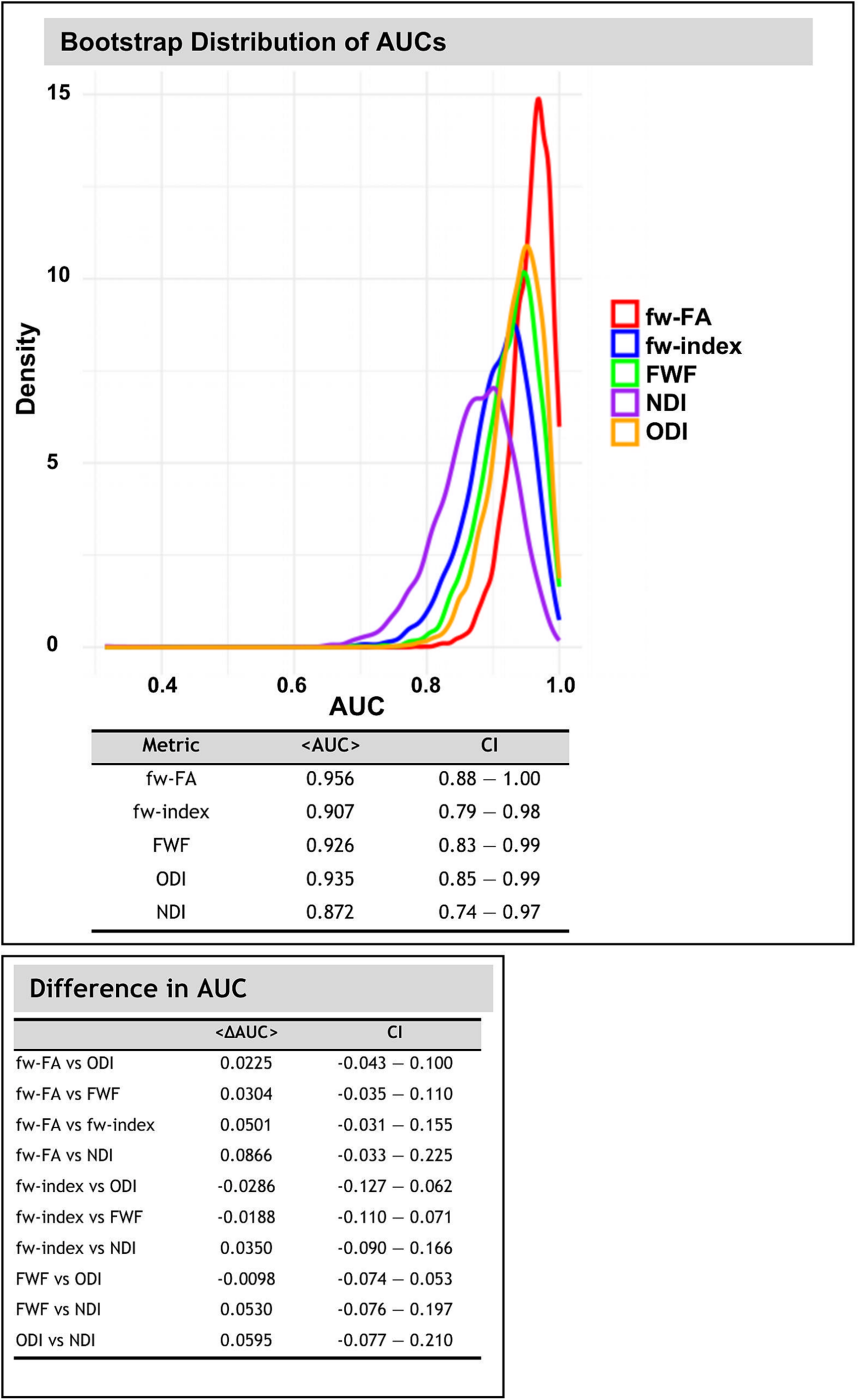
Bootstrap distribution of area under curve (AUC) values (using 10,000 iterations) for fw-DTI and NODDI metrics. The density plot illustrates the distribution of AUC values obtained through bootstrapping for each metric. The accompanying tables summarize the mean AUC values with 95% confidence intervals (CI) and pairwise differences in AUC between metrics.

Figure 7 illustrates the bootstrap distributions of AUC values for fw-DTI and NODDI metrics, along with the pairwise comparisons of AUC differences. The classification algorithm was used to differentiate between HC and PD in OFF conditions. The AUC distributions were calculated within the significant clusters identified after TFCE and FDR correction (*p* < 0.05).

Across all diffusion-related metrics, the highest classification performance was observed for fw-FA, with a mean AUC of 0.956 (95% CI: 0.883–1.000), followed by ODI (AUC = 0.935, 95% CI: 0.847–0.993) and FWF (AUC = 0.926, 95% CI: 0.883–0.992). The fw-index also showed strong discriminative power with an AUC of 0.907 (95% CI: 0.793–0.984), whereas NDI had the lowest AUC at 0.872 (95% CI: 0.742–0.968).

Pairwise comparisons of AUC values revealed differences across the diffusion metrics. The largest differences were found between fw-FA and NDI (<ΔAUC> = 0.085, 95% CI: −0.032 to 0.225).

## 4 Discussion

Today, levodopa remains the most effective treatment for Parkinson’s disease (PD), alleviating many of the motor symptoms. Prolonged use of levodopa leads to motor fluctuations between OFF (withdrawn) and ON (post-levodopa) states, which can significantly impact daily functioning and quality of life (7, 48). Advanced dMRI techniques, such as multi-shell fw-DTI (fw-DTI) (39) and NODDI (22), offer deeper insights into PD-related microstructural alterations beyond conventional DTI. In this study, we applied multi-shell fw-DTI and NODDI to investigate voxel-wise white matter differences in 20 PD patients across OFF and ON states and compared PD (OFF) with a cohort of HC participants. By correlating imaging findings with clinical scores, we aimed to enhance the understanding of PD-related neurodegeneration and levodopa-induced neural changes.

To ensure precise characterization of microstructural alterations, we implemented a robust dMRI processing pipeline and advanced statistical analyses. Traditional single-shell dMRI, which uses only one *b*-value, is constrained by a simplified diffusion model, making it challenging to separate free-water contamination from tissue-specific diffusion properties. In contrast, multi-shell dMRI samples diffusion at multiple *b*-values, enabling more precise separation of the free-water and tissue compartments (39). This improved modeling reduces biases inherent in single-shell methods, leading to more accurate estimates of white matter microstructure and better sensitivity to pathological changes in neurodegenerative diseases like PD (18). Therefore, multi-shell fw-DTI can offer improved sensitivity and specificity in detecting microstructural alterations in neurodegenerative diseases. Additionally, NODDI provides complementary information on neurite density and orientation dispersion, further refining the assessment of PD-related white matter integrity (22).

The use of multi-shell fw-DTI and NODDI was guided by pathophysiological features specific to PD. In PD, free-water accumulation in the posterior substantia nigra—measured via fw-DTI—has been strongly associated with dopaminergic neuron loss (19) and correlates with disease progression (14). Unlike conventional DTI metrics (e.g., FA, MD), which conflate extracellular fluid changes with tissue damage, fw-DTI uses compartmental modeling to isolate free-water effects, enabling earlier detection of neurodegeneration (38). For example, Ofori et al. showed that increases in nigral free-water precede detectable FA changes in early PD, underscoring fw-DTI’s sensitivity to early pathological changes (19).

NODDI, in contrast, is a complementary biophysical model that addresses limitations of traditional DTI and DKI—such as the assumption of Gaussian diffusion and nonspecific metric changes. Several studies support NODDI’s utility for PD diagnosis and tracking disease progression (23, 25, 49). Together, fw-DTI and NODDI overcome the constraints of single-shell DTI, offering enhanced specificity in distinguishing overlapping pathological processes—a key step toward developing stage-specific biomarkers for PD.

In this study, both age and sex were included as a covariate in the statistical analysis to account for potential confounding effects. Our results showed no significant age differences between HC and PD groups, though a significant sex difference was observed. Sex differences have been reported in white matter microstructure, including variations in fractional anisotropy and mean diffusivity (50, 51). By controlling for sex, we ensure that observed group differences in DTI metrics are not driven by underlying sex-related variations in brain structure. Motion parameters were comparable across groups and conditions, ensuring no motion-related bias in imaging analyses.

Significant differences in fw-DTI metrics between HC and PD (OFF) groups indicate widespread microstructural alterations in PD-related white matter pathways. The elevated fw-index in PD (OFF) suggests increased extracellular free water, potentially reflecting neuroinflammatory or neurodegenerative processes. The regions with increased fw-index values included the right cingulum (cingulate gyrus), body of the corpus callosum, and superior corona radiata—regions integral to motor control and cognitive function (52–54). Our results align with previous fw-DTI studies on PD-related white matter alterations. Ofori et al. reported increased free-water content in the posterior substantia nigra, suggesting neurodegenerative changes in this region (14), while Planetta et al. found elevated free-water levels in motor-related pathways correlated with disease severity (55). These findings suggest that fw-DTI could provide a non-invasive biomarker of neurodegenerative changes in PD, especially in white matter regions implicated in motor functions. Clinically, this could support earlier diagnosis or stratification of patients based on disease burden.

Similarly, fw-FA was significantly higher in PD (OFF) across multiple white matter tracts, including the anterior thalamic radiation (bilaterally), left superior longitudinal fasciculus, and anterior limb of the internal capsule. These findings, supported by large effect sizes, may reflect underlying pathological changes or adaptive responses in PD. In contrast, a significant decrease in fw-FA in the left retrolenticular part of the internal capsule highlighted regional variability in PD-associated microstructural changes. Increased FA values in PD compared to HC have also been reported. Mole et al. found significantly higher FA in motor pathways, including the corticospinal and thalamocortical tracts, possibly indicating compensatory neuroplasticity or selective neurodegeneration (56). Additionally, Lenfeldt et al. observed increased FA in the substantia nigra, potentially reflecting gliosis, inflammation, or surrounding fiber intrusion into the shrinking structure (57).

Voxel-based comparisons between PD (OFF) and PD (ON) states revealed higher fw-related metric values in the ON state. However, these differences were confined to small clusters (<1% of the corresponding white matter region) and were not supported by effect size analysis, suggesting limited clinical relevance. The absence of widespread free-water changes following levodopa administration may reflect the short timeframe between OFF and ON states, which is insufficient for large-scale neurochemical or microstructural alterations to manifest. These findings appear consistent with a study by Chung et al., which explored the use of single-shell fw-DTI in PD patients. The study found no significant differences in fw-FA or fw-index between the OFF and ON states following L-DOPA administration. This suggests that fw-DTI may be better suited for evaluating chronic structural changes in PD rather than acute, medication-induced fluctuations (17).

NODDI-derived metrics revealed significant microstructural differences between HC and PD (OFF). Increased FWF in PD (OFF) across multiple white matter regions—particularly the forceps minor, superior longitudinal fasciculus, middle cerebellar peduncle, body of the corpus callosum, and right superior corona radiata—suggested elevated extracellular water, indicative of neuroinflammatory or neurodegenerative processes. Large effect sizes confirmed the robustness of these findings. Mitchell et al. previously compared NODDI and fw-DTI biomarkers in PD and atypical Parkinsonism. Both techniques detected microstructural changes, with fw imaging showing higher effect sizes for extracellular changes (e.g., elevated free water in the substantia nigra). NODDI provided additional insights into intracellular volume and orientation dispersion. Both methods were able to discriminate PD from atypical Parkinsonism, with fw-DTI being more sensitive to extracellular neurodegeneration (24).

Interestingly, the FWF differences observed in the PD OFF *vs.* ON condition were predominantly right-lateralized. While our cohort was mostly right-handed (16 right-handed, 2 left-handed, 2 ambidextrous), information regarding symptom lateralization was not collected for this study. Given that PD often presents with asymmetric motor symptoms and lateralized neurodegeneration, this pattern may reflect underlying hemispheric differences in disease burden. Handedness and symptom asymmetry are potential modulators of such imaging findings, and future studies with detailed clinical lateralization data are warranted to further explore these associations.

The findings from both fw-DTI and NODDI highlight the utility of free-water imaging in PD. Both models identified increased extracellular water in PD (OFF), with FWF (from NODDI) and fw-index (from fw-DTI) showing consistent results across overlapping white matter regions, such as the superior longitudinal fasciculus. This agreement suggests that fw metrics are robust biomarkers for neuroinflammatory or neurodegenerative processes in PD. Additionally, FWF may offer advantages over the fw-index (from multi-shell fw-DTI) due to its ability to disentangle intracellular and extracellular contributions more precisely, providing a more nuanced understanding of tissue microstructure.

In addition to FWF changes, NODDI-based ODI was significantly increased in PD (OFF) in several white matter regions, including the left retrolenticular internal capsule and left sagittal stratum, suggesting greater neurite orientation complexity, possibly due to compensatory mechanisms or altered connectivity. However, regions with lower ODI values in PD (OFF) than HC were also identified, though without strong effect size support, indicating regional variability in disease-related changes.

NDI was significantly lower in PD (OFF), with the most significant reductions in the left anterior thalamic radiation and forceps minor, reflecting decreased neurite density consistent with known PD-related neurodegeneration. Large effect sizes supported the relevance of these findings. Similar to these findings, Kamagata et al. previously reported reduced intracellular volume fraction (similar to NDI) in the substantia nigra pars compacta (SNpc) and putamen, correlating with disease severity (25). Additionally, Wei et al. investigated white matter microstructural alterations in PD patients compared to HCs using NODDI, identifying significant changes in NDI, ODI, and ISO in tracts such as the anterior thalamic radiation and corticospinal tract. These changes correlated with gait impairments, highlighting axonal degeneration’s role in motor deficits (26).

For NODDI metrics, white matter changes were also observed between pre- and post-levodopa. Higher FWF in the ON state, particularly in the right corticospinal tract, left inferior fronto-occipital fasciculus, and right posterior thalamic radiation, suggests transient neurochemical changes or medication-induced fluid regulation shifts. Significant NDI increases in associative and visual processing pathways (e.g., right inferior fronto-occipital and inferior longitudinal fasciculus) could suggest transient neurite density enhancement, possibly due to improved neurotransmission or compensatory mechanisms. However, ODI differences between OFF and ON states were minimal and lacked strong effect sizes, suggesting a limited impact of levodopa on neurite orientation.

Within-subject comparisons revealed a significant improvement in motor function after levodopa administration (via the MDS-UPDRS-III). At the same time, the H&Y score remained unchanged, likely due to its categorical nature and lower sensitivity to short-term motor fluctuations. The voxel-based correlation analysis between fw-DTI/NODDI metrics and MDS-UPDRS-III/H&Y revealed significant associations between white matter microstructure and disease severity in both OFF and ON states. A positive correlation was observed between the fw-index and MDS-UPDRS-III scores in both conditions, suggesting that greater free-water content is associated with more severe motor impairment. Additionally, the volumes of the correlation clusters were reduced in the ON state. Similarly, negative correlations between fw-FA and MDS-UPDRS-III indicate that reduced white matter integrity is linked to worse motor function. These correlations were strongest in the splenium of the corpus callosum, a region implicated in interhemispheric communication and motor coordination. Correlations with the H&Y scores followed a similar pattern, with the fw-index showing positive correlations and fw-FA showing negative correlations in both OFF and ON states, with a reduction in correlation volumes in the ON state.

FWF showed significant positive correlations with MDS-UPDRS-III and H&Y scores in both states, indicating that increased extracellular water content is associated with greater motor impairment. This aligns with the hypothesis that neurodegenerative processes, including neuroinflammation and axonal loss, contribute to worsening PD symptoms. However, these correlations were primarily observed in the OFF state, with a dramatic decrease in significant correlation volume in the ON state. This could indicate that these biomarkers primarily reflect the more chronic state, independent of the temporary improvements induced by levodopa. Orientation dispersion index (ODI) also exhibited positive correlations with both clinical scores, further supporting the idea that increased neurite complexity is associated with disease severity. This could reflect compensatory structural reorganization or maladaptive changes in response to neurodegeneration. In contrast, neurite density index (NDI) was negatively correlated with MDS-UPDRS III and H&Y scores, with lower NDI values linked to more severe motor impairment. This suggests that reduced neurite density, indicative of axonal degeneration, is a key factor in PD progression.

These findings highlight the potential of fw-DTI and NODDI metrics as biomarkers for tracking disease severity and treatment response in PD. The distinct correlation patterns of FWF, ODI, and NDI with clinical scores suggest that multiple microstructural processes contribute to PD pathology, emphasizing the need for multimodal imaging approaches to fully understand the disease’s impact on white matter integrity.

Several studies have examined the relationship between diffusion imaging metrics—DTI, fw-DTI, and NODDI—and motor symptom severity in PD as assessed by the MDS-UPDRS-III. DTI-derived measures, such as FA and mean diffusivity (MD), have been linked to MDS-UPDRS-III scores, suggesting that white matter microstructural alterations are associated with disease severity (58). Fw-DTI studies have shown that increased free-water content in the posterior substantia nigra correlates with higher MDS-UPDRS-III scores, indicating that extracellular water accumulation may contribute to motor impairment (14, 55). NODDI-based analyses have reported significant correlations between orientation dispersion index (ODI) and neurite density index (NDI) with MDS-UPDRS-III, reflecting the role of neurite complexity and axonal degeneration in PD-related motor deficits (26).

The bootstrap analysis of AUC distributions provides important information about the discriminative power of fw-DTI and NODDI metrics in distinguishing between groups. As illustrated in Figure 7, fw-FA exhibited the highest classification performance with an AUC of 0.956, indicating its strong sensitivity in detecting microstructural differences. This was followed by ODI (AUC = 0.935), FWF (AUC = 0.926), and fw-index (AUC = 0.907), all demonstrating robust predictive capability. In contrast, NDI showed the lowest AUC (0.872), suggesting relatively weaker discriminative power in this context. These results indicate that fw-FA and related diffusion metrics have strong potential for clinical use in distinguishing PD patients from healthy individuals. Although currently limited to research settings, these advanced imaging metrics may eventually aid in diagnostic support, particularly if future studies confirm their utility in very early-stage patients and in cases where the diagnosis is uncertain.

Pairwise comparisons of AUC values further highlight differences in classification performance among the metrics. The largest observed difference was between fw-FA and NDI (<ΔAUC> = 0.085), suggesting that free-water-corrected fractional anisotropy provides superior group differentiation compared to neurite density index. However, the CI for all pairwise comparisons include zero, indicating that the observed AUC differences are not statistically significant.

Although the current study did not directly examine gray matter structures such as the SN, previous diffusion MRI studies have reported consistent microstructural alterations in the SN of individuals with PD, including increased FW content and reduced FA (14, 55). These changes are thought to reflect neurodegeneration and gliosis in this key dopaminergic region. While our analyses were restricted to white matter, the observed alterations in diffusion metrics, particularly in projection pathways and frontal tracts, may reflect downstream effects of SN pathology, consistent with the known progression of PD-related neurodegeneration along cortico-subcortical circuits.

The primary limitation of this study is the relatively small sample size, which may reduce statistical power and limit the generalizability of the results. Additionally, the OFF scans were consistently acquired before the ON scans, we cannot fully rule out the potential influence of time-of-day effects on diffusion metrics; however, existing literature suggests such circadian variation may primarily affect CSF or gray matter rather than white matter, and reported findings remain mixed regarding the directionality of these changes (59). There are also well-known limitations of voxel-based analysis that may be overcome with advanced methods such as tract-based spatial statistics (TBSS); however, TBSS may limit sensitivity to white matter changes in the peripheral and boundary white matter regions where fw-DTI and NODDI metrics often show pathological changes. While the high AUC values (see CI of fw-FA) suggest strong classification performance, the modest cohort size raises concerns about potential overfitting, where the model may capture noise rather than biologically meaningful patterns. Replication in larger, independent cohorts is essential to validate these findings, improve the reliability of voxel-based comparisons, and enable robust subgroup analyses. Additionally, while our pipeline involved an initial upsampling of the original 2 mm DTI data to 1.25 mm using mrgrid prior to registration with the 1 mm MNI template, a step taken to optimize subsequent non-linear registration accuracy with ANTs SyN, we acknowledge the theoretical limitation of introducing potential interpolation artifacts during the upsampling process. However, we employed a high-quality interpolation method within mrgrid and visually inspected registration results to mitigate this risk. Future work could explore the impact of direct registration from the native 2 mm resolution or alternative multi-resolution registration strategies. Another limitation is the application of spatial smoothing in our data. While implemented to enhance signal detection and account for inter-subject variability, this smoothing reduced the effective spatial resolution compared to the native 2 mm in-plane acquisition. Consequently, fine anatomical details may have been blurred, and highly focal microstructural differences smaller than the smoothing kernel could have been averaged out.

## 5 Conclusion

This study provides new insights into PD-related white matter alterations using advanced diffusion MRI techniques, including multi-shell fw-DTI and NODDI. Our findings highlight significant microstructural differences between PD patients and HCs, particularly in free-water content, neurite density, and orientation dispersion, suggesting widespread neurodegenerative changes beyond the basal ganglia. Additionally, intra-individual comparisons between OFF and ON medication states revealed regionally constrained effects of levodopa on white matter microstructure, emphasizing the complexity of dopaminergic treatment responses. Correlations between diffusion metrics and MDS-UPDRS-III/H&Y further reinforce the role of white matter integrity in motor impairment. This study underscores the potential of fw-DTI and NODDI as sensitive biomarkers for tracking disease progression and treatment effects in PD.

## Data Availability

The datasets and analysis tools generated and used in this study are not publicly available but can be made available from the corresponding author upon reasonable request.

## Footnotes

^1^ https://www.parkinsonsmeasurement.org/toolBox/levodopaEquivalentDose.htm

^2^ http://stnava.github.io/ANTs/

^3^ https://github.com/daducci/AMICO
