# Supplementary material for: Investigating white matter alterations in Parkinson’s disease using multi-shell free-water DTI and NODDI: insights into neurodegeneration and levodopa effects
Source: Front Neurol. 2025 Jul 9;16:1605753. doi: 10.3389/fneur.2025.1605753 (PMC12283281; doi:10.3389/fneur.2025.1605753)

Supplementary Material

Supplementary Figure 1: The violin plots illustrate the distribution of fw-index and fw-FA values for HC and PD (OFF) (Panels (a) and (b)) and for PD (OFF) and PD (ON) (Panels (c) and (d)) highlighting statistical differences and effect sizes.

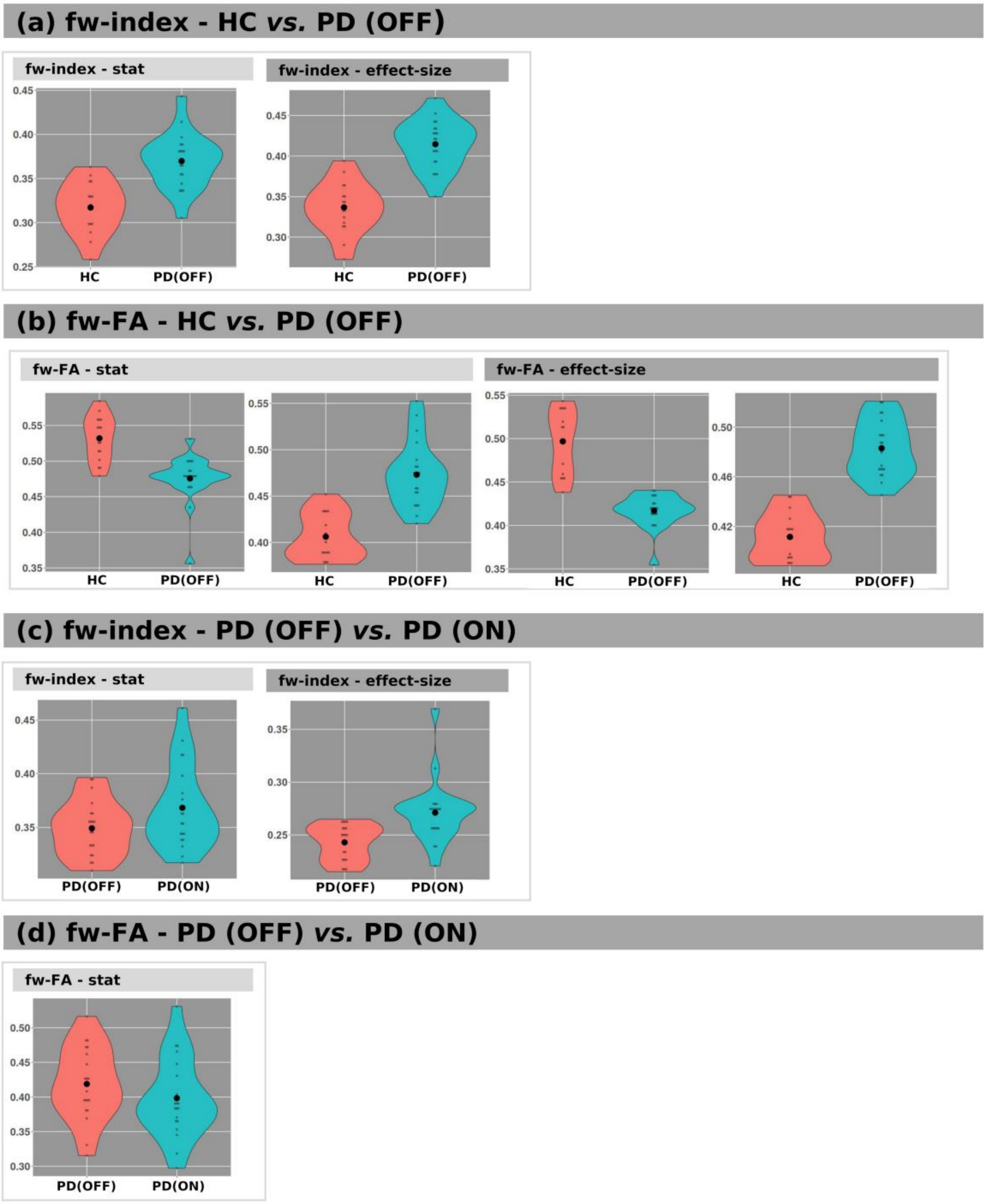

Supplementary Figure 2: The violin plots illustrate the distribution of NODDI metrics for HC and PD (OFF) (Panels (a), (b) and (c)) and for PD (OFF) and PD (ON) (Panels (d), (e), and (f)) highlighting statistical differences and effect sizes.

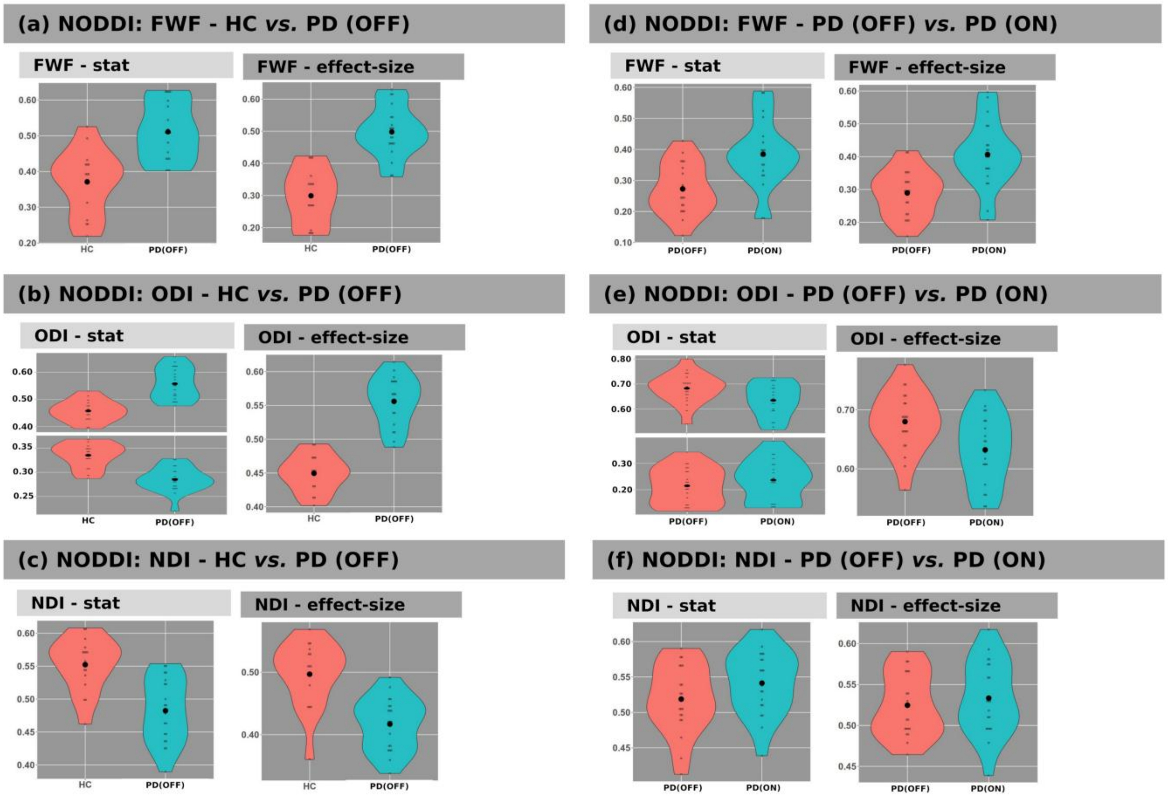

Supplement: Supplementary file 1 [file Data_Sheet_1.pdf]
